# Meroterpenoids With Protein Tyrosine Phosphatase 1B Inhibitory Activities From the Fruiting Bodies of *Ganoderma ahmadii*

**DOI:** 10.3389/fchem.2020.00279

**Published:** 2020-04-16

**Authors:** Jiaocen Guo, Fandong Kong, Qingyun Ma, Qingyi Xie, Renshuai Zhang, Haofu Dai, Yougen Wu, Youxing Zhao

**Affiliations:** ^1^Hainan Key Laboratory for Research and Development of Natural Product From Li Folk Medicine, Institute of Tropical Bioscience and Biotechnology, Chinese Academy of Tropical Agriculture Sciences, Haikou, China; ^2^College of Horticulture, Hainan University, Haikou, China; ^3^Qingdao Cancer Institute, The Affiliated Hospital of Qingdao University, Qingdao, China

**Keywords:** *Ganoderma ahmadii*, meroterpenoid, spectroscopic, PTP1B, cytotoxicity

## Abstract

*Ganoderma* fungi have long been used as functional foods and traditional medicines in Asian countries. *Ganoderma ahmadii* is one of the main species of *Ganoderma* fungi distributed in Hainan province of China, the fruiting bodies of which have been used in folk to lower blood sugar for a long time. A chemical investigation of the fruiting bodies of *Ganoderma ahmadii* led to the isolation of seven new meroterpenoids, named ganoduriporols F-L **(1–7)**. The chemical structures of the compounds were elucidated by spectroscopic data including HRESIMS and 2D NMR. Compounds **5–7** represent the first examples of ganoduriporol-type meroterpenoids bearing oxepane rings in their skeletons. Compounds **1–4** showed inhibitory activity against protein tyrosine phosphatase 1B (PTP1B) comparable to the positive control Na_3_VO_4_, with IC_50_ values of 17, 20, 19, and 23 μM, respectively.

## Introduction

*Ganoderma* fungi have been widely used as functional foods and traditional medicines, which have provided more efficient means for human health care, nutrition, medical care in China (Ma et al., 2019). It has been regarded as one of the most important medicinal fungi for preventing and treating various human diseases in Asian countries (Paterson, 2006). Previous studies have showed that the bioactive constituents of these fungi are mainly triterpenoids (Baby et al., 2015), polysaccharides (Wang et al., 2014), alkaloids (Zhao et al., 2015), and meroterpenoids (Yan et al., 2013) etc. These compounds with diverse structures displayed various biological effects, such as anti-tumor (Fu et al., 2019), anti-inflammatory (Lu et al., 2019), anti-diabetes (Wang et al., 2017), immunomodulation (Ji et al., 2007), and anti-oxidation activities (Qiu et al., 2016). Recently, a great deal of work on *Ganoderma* fungi have found that some constituents extracted from *Ganoderma* can promote the release of serum insulin and decrease the plasma glucose concentration *in vivo* (Huang et al., 2010; Li et al., 2017; Zhao and He, 2018).

Recent studies on the pathological mechanism revealed that type 2 diabetes has a close relationship with the protein tyrosine phosphatase family, which plays an important role in the negative regulator of insulin signaling by dephosphorylating the tyrosine residues of proteins (Tamrakar et al., 2014). PTP1B is an important member of the protein tyrosine phosphatase family and is responsible for insulin signaling pathway (Wang et al., 2015). Insulin resistance caused by expression of PTP1B as well as dephosphorylation of its target is one of the main causes of type 2 diabetes (Cai et al., 2015). Thus, PTP1B has been identified as a target for research and development of new drugs for the treatment of type 2 diabetes, and PTP1B inhibitors are potential lead compounds for such new drugs (Teng et al., 2011).

*Ganoderma ahmadii* is mainly distributed in Hainan, Yunnan, and Guizhou provinces in China (Wu and Dai, 2005), which have been used in folk medicine to lower blood sugar for a long time. As our ongoing search for bioactive constituents from the genus *Ganoderma* (Zhang et al., 2015; Huang et al., 2016, 2017), the bioactive constituents from *G. ahmadii* was studied, which led to the isolation of three new meroterpenoids with PTP1B inhibitory activity (Guo et al., 2019). A continuous research resulted in the isolation of another seven new meroterpenoids, named ganoduriporols F-L (**1–7**). Herein, the isolation, structural characterization, and PTP1B inhibitory activities of these compounds are reported.

## Materials and Methods

### General Experimental Procedures

The NMR spectra were recorded on a Bruker AV-500 spectrometer (Bruker, Bremen, Germany), and using tetramethylsilane (TMS) as an internal standard. Chemical shifts (δ) were expressed in ppm with reference to TMS. High Resolution Electrospray Ionization Mass Spectroscopy (HRESIMS) data were acquired using a mass spectrometer API QSTAR Pulsar (Bruker, Bremen, Germany). Optical rotations were measured using a JASCO P-1020 digital polarimeter. UV spectra were obtained with a Beckman DU 640 spectrophotometer. IR spectra were recorded on with a Shimadzu UV2550 spectrophotometer (Japan). Semipreparative high-performance liquid chromatography (HPLC) equipped with octadecyl silane (ODS) column (COSMOSIL-pack ODS-A, 10 × 250 nm, 5 μm, 4 ml/min) and phenyl (PH) column (COSMOSIL-pack ph, 10 × 250 nm, 5 μm, 4 ml/min) were used to isolate compounds. Silica gel (200-300 mesh; Qingdao Marine Chemical Inc., Qingdao, China) and Sephadex LH-20 (40–70 μm; Merck, Darmstadt, Germany) were used for column chromatography. Thin-layer chromatography (TLC) was carried out with precoated Si gel plates.

### Plant Material

The *Ganoderma ahmadii* were collected in June 2017 Qiongzhong County, Hainan Province, China. The fungal material was identified by Prof. Zeng Nian-Kai (Hainan Medical University, China). The certified specimen (No.011-ZLZ) was deposited in the Institute of Tropical Bioscience and Biotechnology, Chinese Academy of Tropical Agricultural Sciences.

### Extraction and Isolation

The dried and powdered fruiting bodies of *G. ahmadii* (5.0 kg) were extracted with 95% ethanol three times at room temperature. After filtration and evaporation, a gummy residue was obtained, which was taken up in H_2_O and with petroleum ether, ethyl acetate (EtOAc), and n-butanol. The EtOAc extract (55.0 g) was subjected to column chromatography (CC) on silica gel with gradient elution (petroleum ether-EtOAc, 8:1–1:2), which yielded seven fractions (Fr.1–Fr.7). Fr.6 (5.0 g) was further separated using an octadecyl silane silica gel column and eluted with gradient solvent of MeOH-H_2_O (30–100%) to give seven fractions (Fr.6.1–Fr.6.7), Fr.6.5 (116.0 mg) was purified by semipreparative HPLC [42% MeCN/H_2_O, containing 0.1% trifluoroacetic acid (TFA)] to afford compound **2** (*t*_R_ 15.0 min; 3.5 mg). Fr.6.6 (140.0 mg) was further purified using semipreparative HPLC (70% MeOH/H_2_O, containing 0.1% TFA) to give compound **4** (*t*_R_ 12.2 min; 3.0 mg). Fr.7 (6.0 g) was subjected to CC on an ODS elution with MeOH-H_2_O (20–100%) to give nine fractions (Fr.7.1–Fr.7.9). Fr.7.1 (2.2 g) was subjected to Sephadex LH-20 eluting with CHCI_3_-MeOH-petroleum ether (1:1:1) yielded three sub-fractions (Fr.7.1.1–7.1.3) based on TLC. Fr.7.1.2 (87.0 mg) was further purified by semipreparative HPLC (45% MeCN/H_2_O; containing 0.1% TFA) to afford compound **3** (*t*_R_ 7.8 min; 3.0 mg). Fr.7.1.3 (70.0 mg) was subjected to semipreparative HPLC (65% MeOH/H_2_O; containing 0.1% TFA) to afford compound **6** (*t*_R_ 32.0 min; 3.0 mg). Fr.7.2 (150.0 mg) was further purified by semipreparative HPLC (45% MeCN/H_2_O; containing 0.1% TFA) to afford compounds **7** (*t*_R_ 20.7 min; 3.3 mg) and **5** (*t*_R_ 22.2 min; 3.0 mg). Fr.7.7 (1.5 g) was subjected to Sephadex LH-20 (MeOH) to afford five fractions (Fr.7.7.1–Fr.7.7.5), in which Fr.7.7.4 (80.0 mg) was further prepared by semipreparative HPLC (72% MeOH/H_2_O, containing 0.1% TFA) to produce compound **1** (*t*_R_ 14.2 min; 3.5 mg).

### Characterization of Compounds 1–7

Ganoduriporol F (**1**): yellow oil; UV (MeOH) λ max (log ε) 307 (4.4), 224 (4.0) nm; IR (KBr) ν_max_ (cm^−1^): 3436, 2925, 2851, 1630, 1388, 1168 (Figure S9); ^1^H and ^13^C NMR data, see Tables 1, 2; HRESIMS *m/z* 559.1938 [M + Na]^+^ (calcd for C_30_H_32_NaO_9_, 559.1939).

**Table 1:** ^13^C NMR (125 MHz) Data of Compounds **1–7** in CD_3_OD (δ in ppm).

**NO**.	**1**	**2**	**3**	**4**	**5**	**6**	**7**
1	157.1, C	157.2, C	156.6, C	157.2, C	156.6, C	156.6, C	156.6, C
2	121.4, C	121.3, C	120.4, C	121.3, C	121.0, C	121.1, C	120.9, C
3	115.8, CH	115.8, CH	115.7, CH	115.8, CH	116.0, CH	116.1, CH	115.9, CH
4	150.7, C	150.8, C	150.7, C	150.8, C	150.6, C	150.5, C	150.6, C
5	126.7, CH	126.7, CH	125.9, CH	126.7, CH	126.0, CH	126.0, CH	126.0, CH
6	119.9, CH	120.0, CH	119.7, CH	120.0, CH	119.6, CH	119.6, CH	119.7, CH
1′	198.6, C	198.8, C	204.3, C	198.7, C	204.1, C	204.4, C	203.9, C
2′	132.9, CH	132.7, CH	37.5, CH_2_	132.8, CH	46.4, CH_2_	47.1, CH_2_	45.3, CH_2_
3′	146.0, C	146.4, C	127.7, C	146.6, C	82.0, C	81.6, C	82.1, C
4′	29.5, CH_2_	29.6, CH_2_	147.0, CH	29.4, CH_2_	22.1, CH_2_	22.5, CH_2_	24.5, CH_2_
5′	28.1, CH_2_	27.5, CH_2_	27.3, CH_2_	27.3, CH_2_	38.4, CH_2_	37.6, CH_2_	36.2, CH_2_
6′	127.6, CH	31.6, CH_2_	31.7, CH_2_	31.9, CH_2_	32.5, CH_2_	36.7, CH_2_	126.0, CH
7′	140.3, C	40.7, CH	37.8, CH	37.5, CH	41.4, CH	42.1, CH	140.8, C
8′	35.0, CH_2_	31.3, CH_2_	31.0, CH_2_	31.6, CH_2_	36.4, CH_2_	32.7, CH_2_	36.5, CH_2_
9′	27.2, CH_2_	25.9, CH_2_	25.8, CH_2_	25.7, CH_2_	26.3, CH_2_	26.2, CH_2_	27.4, CH_2_
10′	130.2, CH	130.8, CH	130.1, CH	130.3, CH	130.5, CH	130.4, CH	129.5,CH
11′	131.5, C	131.5, C	132.0, C	131.9, C	131.2, C	131.7, C	132.0, C
12′	71.1, CH_2_	71.2, CH_2_	71.0, CH_2_	71.0, CH_2_	71.1, CH_2_	71.1, CH_2_	70.9, CH_2_
13′	14.1, CH_3_	14.1, CH_3_	14.1, CH_3_	14.1, CH_3_	14.0, CH_3_	14.1, CH_3_	14.1, CH_3_
14′	172.7, C	170.0, C	170.4, C	169.9, C	178.0, C	178.2, C	177.1, C
15′	59.9, CH_2_	65.4, CH_2_	67.5, CH_2_	67.9, CH_2_	69.2, CH_2_	70.9, CH_2_	66.8, CH_2_
16′			173.0, C	173.2, C			
17′			20.7, CH_3_	20.8, CH_3_			
1″	169.3, C	169.3, C	169.1, C	169.2, C	169.2, C	169.3, C	169.1, C
2″	115.1, CH	115.1, CH	115.2, CH	115.1, CH	115.2, CH	115.1, CH	115.1, CH
3″	146.6, CH	146.9, CH	146.6, CH	146.6, CH	146.5, CH	146.6, CH	146.6, CH
4″	127.1, C	127.7, C	127.1, C	127.1, C	127.1, C	127.1, C	127.1, C
5″	131.2, CH	115.1, CH	131.2, CH	131.2, CH	131.2, CH	131.2, CH	131.2, CH
6″	116.8, CH	146.8, C	116.8, CH	116.8, CH	116.8, CH	116.8, CH	116.8, CH
7″	161.1, C	149.6, C	161.2, C	161.2, C	161.3, C	161.2, C	161.3, C
8″	116.8, CH	116.5, CH	116.8, CH	116.8, CH	116.8, CH	116.8, CH	116.8, CH
9″	131.2, CH	123.0, CH	131.2, CH	131.2, CH	131.2, CH	131.2, CH	131.2, CH

**Table 2:** ^1^H NMR (500 MHz) Data of Compounds **1-7** in CD_3_OD (δ in ppm, *J* in Hz).

**NO**.	**1**	**2**	**3**	**4**	**5**	**6**	**7**
3	7.13, d (2.9)	7.11, d (2.7)	7.34, d (2.8)	7.09, d (2.7)	7.25, d (3.0)	7.29, d (2.8)	7.23, d (2.9)
5	7.05,dd (9.0, 2.9)	7.01, dd (8.9, 2.7)	6.99, dd (8.9, 2.8)	7.09, dd (8.9, 2.7)	6.99, dd (8.9, 3.0)	7.04, dd (8.9, 2.8)	6.98, dd (9.0, 2.9)
6	6.84, d (9.0)	6.80, d (8.9)	6.78, d (8.9)	6.79, d (8.9)	6.77, d (8.9)	6.80, d (8.9)	6.75, d (9.0)
2′	7.70, s	7.65, s	4.06, s	7.64, s	3.64, m 3.30, overlap	3.73, overlap 3.24, m 3.24 (m)	3.77, m 3.33, m
4′	2.65, t (7.5)	2.54, t (7.5)	2.22, t (7.7)	2.52, t (7.5)	1.75, overlap 1.57, m	1.79, m 1.72, m	2.28, overlap 2.21, m
5′	2.31, m	1.51, m	2.22, m	1.58, m	2.19, m 2.03, overlap	2.13, m	2.28, overlap 2.17, m
6′	5.26, t (7.7)	1.39, overlap 1.27, overlap	1.49, overlap 1.38, overlap	1.31, m	1.33, m 1.21, overlap	1.87, m 1.14, m	5.43, overlap
7′		1.39, overlap	1.68, m	1.50, m	1.68, m	1.61, m	
8′	2.07, overlap	1.39, overlap 1.27, overlap	1.49, overlap 1.38, overlap	1.31, m	1.74, overlap 1.21, overlap	1.31, m 1.24, m	1.96, overlap
9′	2.10, overlap	2.03, m	2.08, m	2.03, m	2.03, overlap	2.09, m	2.10, m 1.97, overlap
10′	5.43, t (6.7)	5.45, t (7.2)	5.44, t (7.3)	5.43, t (7.2)	5.46, t (7.3)	5.50, t (7.3)	5.43, overlap
12′	4.53, s	4.51, s	4.52, s	4.50, s	4.53, s	4.57, s	4.53, s
13′	1.65, s	1.65, s	1.65, s	1.64, s	1.63, s	1.69, s	1.65, s
15′	4.07, s	3.41, m	4.00, m	3.93, m	3.59, m	3.73, overlap 3.55, m	4.23, m 4.17, m
17′			1.96, s	1.96, s			
2″	6.31, d (15.9)	6.23, d (16.0)	6.31, d (15.9)	6.28, d (15.8)	6.32, d (16.0)	6.35, d (16.0)	6.31, d (15.9)
3″	7.60, d (15.9)	7.49, d (16.0)	7.58, d (15.9)	7.55, d (15.8)	7.59, d (16.0)	7.62, d (16.0)	7.58, d (15.9)
5″	7.44, d (8.4)	7.01, s	7.43, d (8.3)	7.40, d (8.1)	7.44, d (8.5)	7.48, d (8.6)	7.42, d (8.5)
6″	6.80, d (8.4)		6.78, d (8.3)	6.74, d (8.1)	6.79, d (8.5)	6.83, d (8.6)	6.77, d (8.5)
8″	6.80, d (8.4)	6.78, d (8.3)	6.78, d (8.3)	6.74, d (8.1)	6.79, d (8.5)	6.83, d (8.6)	6.77, d (8.5)
9″	7.44, d (8.4)	6.90, d (8.3)	7.43, d (8.3)	7.40, d (8.1)	7.44, d (8.5)	7.48, d (8.6)	7.42, d (8.5)

Ganoduriporol G (**2**): yellow oil; ${\left[\alpha \right]}_{\text{D}}^{25}$ +12 (*c* 0.1, MeOH); UV (MeOH) λ _max_ (log ε) 328 (4.1), 288 (3.8) nm; IR (KBr) ν_max_ (cm^−1^): 3414, 2927, 2859, 1610, 1474, 1263, 1195 (Figure S18); ^1^H and ^13^C NMR data, see Tables 1, 2; HRESIMS *m/z* 577.2046 [M + Na]^+^ (calcd for C_30_H_34_NaO_10_, 577.2044).

Ganoduriporol H (**3**): yellow oil; ${\left[\alpha \right]}_{\text{D}}^{25}$ +10 (*c* 0.1, MeOH); UV (MeOH) λ _max_ (log ε) 311 (4.3), 259 (4.0), 206 (4.8) nm; IR (KBr) ν_max_ (cm^−1^): 3418, 2927, 2851, 1688, 1606, 1511, 1389, 1170 (Figure S27); ^1^H and ^13^C NMR data, see Tables 1, 2; HRESIMS *m/z* 603.2212 [M + Na]^+^ (calcd for C_32_H_36_NaO_10_, 603.2201).

Ganoduriporol I (**4**): yellow oil; ${\left[\alpha \right]}_{\text{D}}^{25}$ +17 (*c* 0.1, MeOH); UV (MeOH) λ _max_ (log ε) 292 (4.1), 247 (3.8) nm; IR (KBr) ν_max_ (cm^−1^): 3435, 2926, 2851, 1685, 1603, 1449, 1268, 1169 (Figure S36); ^1^H and ^13^C NMR data, see Tables 1, 2; HRESIMS *m/z* 603.2169 [M + Na]^+^ (calcd for C_32_H_36_NaO_10_, 603.2201).

Ganoduriporol J (**5**): yellow oil; ${\left[\alpha \right]}_{\text{D}}^{25}$ +8 (*c* 0.1, MeOH); UV (MeOH) λ _max_ (log ε) 299 (4.2), 264 (4.0), 225 (4.5) nm; IR (KBr) ν_max_ (cm^−1^): 3418, 2927, 2855, 1691, 1604, 1512, 1271, 1170 (Figure S45); ^1^H and ^13^C NMR data, see Tables 1, 2; HRESIMS *m/z* 561.2065 [M + Na]^+^ (calcd for C_30_H_34_NaO_9_, 561.2095).

Ganoduriporol K (**6**): yellow oil; ${\left[\alpha \right]}_{\text{D}}^{25}$ +10 (*c* 0.1, MeOH); UV (MeOH) λ _max_ (log ε) 312 (4.0), 224 (4.3) nm; IR (KBr) ν_max_ (cm^−1^): 3422, 2928, 2847, 1682, 1602, 1474, 1280, 1198 (Figure S54); ^1^H and ^13^C NMR data, see Tables 1, 2; HRESIMS *m/z* 561.2147 [M + Na]^+^ (calcd for C_30_H_34_NaO_9_, 561.2095).

Ganoduriporol L (**7**): yellow oil; ${\left[\alpha \right]}_{\text{D}}^{25}$ +14 (*c* 0.1, MeOH); UV (MeOH) λ _max_ (log ε) 299 (4.4), 263 (4.1) nm; IR (KBr) ν_max_ (cm^−1^): 3418, 2928, 1693, 1602, 1512, 1391, 1170 (Figure S63); ^1^H and ^13^C NMR data, see Tables 1, 2; HRESIMS *m/z* 559.1918 [M + Na]^+^ (calcd for C_30_H_32_NaO_9_, 559.1939).

### PTP1B Inhibition Assay

The expressed and purified methods of recombinant PTP1B catalytic domain were the same as references (Liu et al., 2018). The dephosphorylation of *p*-nitrophenyl phosphate (*p*NPP) generated the product pNP, which can be monitored at an absorbance for 405 nm. The compounds were pre-incubated with the enzyme at 37 °C for 5 min. Assay was performed in the final volume of 100 μL in the active system containing 50 μL reaction buffer, 25.5 μL double distilled water, 2 μL test compounds and 10 μL enzymes. After incubation at 37°C for 15 min, the reaction was terminated using 0.5 M *p*NPP (12.5 μL). After initializing the enzymatic reaction, the plate was then read every 20 s for 15 min in the microplate reader at 405 nm. Sodium orthovanadate (Na_3_VO_4_) was used as a positive control. The equation used was: [(A_blank_-A_sample_)/A_blank_] × 100%. The IC_50_ values were determined by linear or non-linear regression analysis of the concentration-response data curve.

### Cytotoxicity Assay

All isolated compounds **1-7** were evaluated for their cytotoxicity against BEL, K562, SGC7901, A549 and Hela cell lines. The cytotoxic activities were assayed by using the MTT method in 96 well plates according to the previous report (Shi et al., 2008).

### Molecular Docking

Docking simulation referred to the published literature (Zhang et al., 2017). It was carried out by means of the SYBYL-X 2.0 software. All the ligand molecular were drawn using the standard parameters of SYBYL-X, then their geometric conformations were energy minimized employing the Tripos force field for 1,000 steps and Gasteiger-Huckel charges were calculated. Protein receptor (**PDB: 1QXK**) was prepared using the standard way. The H-bonds were shown using dotted line. Pymol was used as a viewer for interaction between ligands and protein receptor.

## Results and Discussion

### Identification of Compounds 1-7

**Figure 1:**
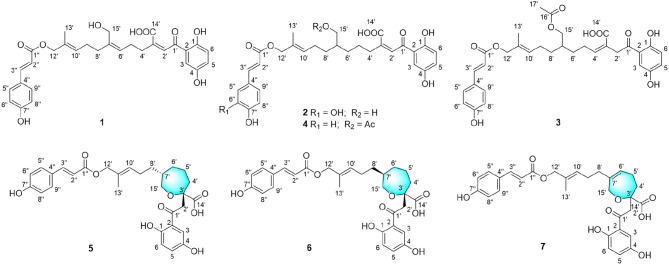
Structures of compounds **1–7**.

**Figure 2:**
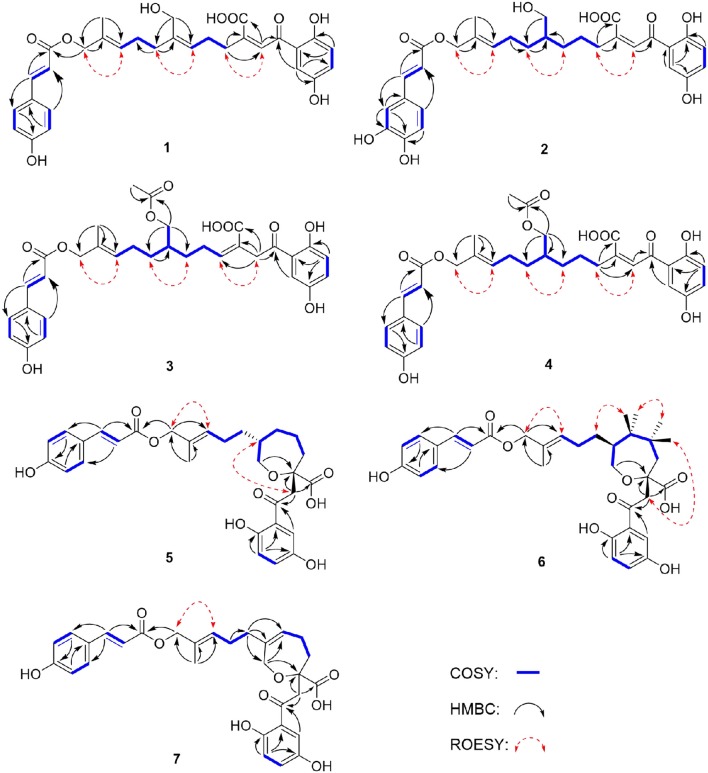
Key COSY, HMBC, and ROESY correlations of compounds **1–7**.

Compound **1** was obtained as yellow oil, with a molecular formula of C_30_H_32_O_9_ from the molecular ion peak [M + Na]^+^ at *m/z* 559.1938 (calcd 559.1939) in the HRESIMS (Figure S8). The ^1^H NMR revealed diagnostic signals of a 2-substituted-1,4-dihydroxylbenzene moiety (δ_H_ 7.13, 7.05, and 6.84), a *p*-substituted hydroxybenzene substructure (δ_H_ 7.44 and 6.80), three olefinic singlets (δ_H_ 7.70, 5.26, and 5.43), two conjugated olefinic doublets (δ_H_ 6.31 and 7.60), and one methyl (δ_H_ 1.65). The ^13^C NMR and the DEPT spectra (Table 1, Figures S2, S3) showed a total of 30 carbon signals including one methyl, six methylenes with two oxygenated, twelve sp^2^ methines, and eleven sp^2^ quaternary carbons including three carboxylic or carbonyl carbons. Comparison of its ^1^H and ^13^C NMR spectral data (Tables 1, 2, Figures S1–S6) with those of ganoduriporol A (Chen et al., 2017) suggested that **1** had a similar structure to ganoduriporol A. Their main structural difference was that the ${\text{CH}}_{2}-2^{\prime }$/CH-3′ substructure in ganoduriporol A was dehydrogenated to form an tri-substituted double bond in **1**, as suggested by heteronuclear multiple bond coherence spectroscopy (HMBC) correlations from the olefinic proton H-2′ (δ_H_ 7.70) to C-3′ (δ_C_ 146.0), C-4′ (δ_C_ 29.5), and C-1′ (δ_C_ 198.6). The configurations of the double bonds as 2′*Z*, 6′*Z*, 10′*E*, and 2″*E* were supported by rotating frame overhauser effect spectroscopy (ROESY) correlations (Figures 1, 2, Figure S7) of H-2′/H_2_-4′ (δ_H_ 2.65), H-6′ (δ_H_ 5.26)/H_2_-8′ (δ_H_ 2.07), H-10′ (δ_H_ 5.43)/H_2_-12′ (δ_H_ 4.53), and H-2″ (δ_H_ 6.31)/H-9″ (δ_H_ 7.44), respectively.

Compound **2** was obtained as yellow oil with a molecular formula of C_30_H_34_O_10_ according to the HRESIMS data (Figure S17). Comparison of the NMR data (Tables 1, 2, Figures S10–S15) of **2** with those of **1** revealed the presence of an additional hydroxyl at C-6″ (δ_C_ 146.8) in **2**, which was further confirmed by the HMBC correlations of H-5″ (δ_H_ 7.01) and H-8″ (δ_H_ 6.78) to C-6″ (δ_C_ 146.8). Besides, the presence of CH_2_-6′/CH-7′ fragment in **2** instead of olefinic double bond CH-6′/C-7′ as in **1** was revealed by HMBC correlations from H_2_-15′ (δ_H_ 3.41) to C-6′ (δ_C_ 31.6), C-7′ (δ_C_ 40.7), and C-8′ (δ_C_ 31.3) (Figures 1, 2). The configurations of 2′*Z*, 10′*E* and 2″*E* in compound **2** were revealed by ROESY correlations (Figure 2, Figure S16) of H-2′ (δ_H_ 7.65)/H_2_-4′ (δ_H_ 2.54), H-10′ (δ_H_ 5.45)/H_2_-12′ (δ_H_ 4.51), and H-2″ (δ_H_ 6.23)/H-5″.

Compound **3** was obtained as yellow oil, and its molecular formula C_32_H_36_O_10_ was concluded from the HRESIMS (Figure S26) *m/z* 603.2212 ([M + Na]^+^, calcd 603.2201), implying 15 degrees of unsaturation. Its ^1^H NMR and ^13^C NMR data (Tables 1, 2, Figures S19–S24) were very similar to those of ganoduriporol B (Chen et al., 2017) with the main difference being the presence of signals for an acetyl group, which located at C-15′ (δ_C_ 67.5) as suggested by HMBC correlations (Figures 1, 2) from H_2_-15′ (δ_H_ 4.00) and a singlet methyl proton signal at δ_H_ 4.00 to the ester carbonyl at δ_C_ 173.0. Besides, the CH-3′/CH_2_-4′ substructure in ganoduriporol B was dehydrogenated to afford an olefinic C-3′/CH-4′ double bond in **3**, as revealed by HMBC correlations from ${\text{H}}_{2}-2^{\prime }$ (δ_H_ 4.06) to C-1′ (δ_C_ 204.3), C-3′ (δ_C_ 127.7), C-4′ (δ_C_ 147.0), and C-14′ (δ_C_ 170.4). The configurations of 3′*Z*, 10′*E* and 2″*E* in compound **3** were revealed by the ROESY correlations (Figure 2, Figure S25) of ${\text{H}}_{2}-2^{\prime }$/H-4′ (δ_H_ 2.22), H-10′ (δ_H_ 5.44)/H_2_-12′ (δ_H_ 4.52), and H-2″ (δ_H_ 6.31)/H-5″ (δ_H_ 7.43).

Compound **4** was isolated as yellow oil. Its molecular formula was determined as C_32_H_36_O_10_ by HRESIMS (Figure S35), the same as that of **3**. The NMR data of **4** were quite similar to those of **3** (Tables 1, 2). Analysis of the 2D NMR data (Figure 2, Figures S28–S33) of **4** suggested their only structural difference was the position of one double bond, which was located C-2′ (δ_C_ 132.8) and C-3′ (δ_C_ 146.6) in **4** instead of at C-3′ (δ_C_ 127.7) and C-4′ (δ_C_ 147.0) as in **3**, as confirmed by HMBC correlations from the olefinic proton H-2′ (δ_H_ 7.64) to C-3′ (δ_C_ 146.6), C-4′ (δ_C_ 29.4), and C-1′ (δ_C_ 198.7). The configurations of 2′*Z*, 10′*E*, and 2″*E* in compound **4** were elucidated by ROESY correlations (Figures 1, 2, Figure S34) of H-2′/H_2_-4′ (δ_H_ 2.52), H-10′ (δ_H_ 5.43)/H_2_-12′ (δ_H_ 4.50), and H-2″ (δ_H_ 6.28)/H-9″ (δ_H_ 7.40).

Compound **5** was assigned the molecular formula as C_30_H_34_O_9_ on the basis of HRESIMS data (Figure S44), indicating 14 degrees of unsaturation. The ^1^H NMR and ^13^C NMR, together with ^1^H-detected heteronuclear single quantum coherence spectrum (HSQC), revealed the presence of one methyl, eight methylenes (two oxygenated), eleven methines, and ten quaternary carbons. Comparison of NMR data (Figures S37–S42) between compounds **5** and **1** found that the Δ^2^′ double bond in **1** was replaced by a methylene (δ_C/H_ 69.2/3.59) and an oxygenated quaternary carbon (δ_C_ 82.0), the latter of which was linked to C-2′ (δ_C_ 46.4) via an oxygen atom to form an oxepane rings, as suggested by HMBC correlations from ${\text{H}}_{2}-2^{\prime }$ (δ_H_ 3.64) to C-3′ (δ_C_ 82.0), C-14′ (δ_C_ 178.0), and C-1′ (δ_C_ 204.1) and from H_2_-15′ (δ_H_ 3.59) to C-3′. The other difference was that the double bond at C-6′ (δ_C_ 127.6) and C-7′ (δ_C_ 140.3) in **1** was replaced by a sp^3^ methine and methylene in **5**, as confirmed by COSY correlations (Figures 1, 2) of H-7′ (δ_H_ 1.68) with H_2_-6′ (δ_H_ 1.21), H_2_-8′ (δ_H_ 1.74) and H_2_-15′. The *E* configurations of Δ^10^′ and Δ^2^″ double bonds were established by the ROESY correlations (Figure 2, Figure S43) from H-10′ (δ_H_ 5.46) to H_2_-12′ (δ_H_ 4.53) and the coupling constant (*J* = 16.0 Hz) between H-2″ (δ_H_ 6.32) and H-3″ (δ_H_ 7.59), respectively. ROESY correlations from ${\text{H}}_{2}-2^{\prime }$ (δ_H_ 3.64)/H-7′ (δ_H_ 1.68) suggested that ${\text{CH}}_{2}-2^{\prime }$ and CH_2_-8′ were on the face opposite to each other.

Compound **6** was determined to have a molecular formula C_30_*H*_34_*O*_9_ based on HRESIMS (Figure S53) analysis, the same as that of **5**. The 1D NMR data of **6** were almost identical to those of **5**. Analysis of the 2D NMR data (Figures S46–S51) of **6** found that compounds **5** and **6** shared the same planar structure, indicating that they are a pair of stereoisomers. The *E* configuration of Δ^10^′ and Δ^2^″ double bonds were established by the ROESY correlations (Figures 1, 2, Figure S52) from H-10′ (δ_H_ 5.50) to ${\text{H}}_{2}-12^{\prime }$ (δ_H_ 4.57) and the coupling constant (*J* = 16.0 Hz) between H-2″ (δ_H_ 6.35) and H-3″ (δ_H_ 7.62), respectively. ROESY correlations from ${\text{H}}_{2}-2^{\prime }$ (δ_H_ 3.24)/H_2_-4′α (δ_H_ 1.72), and H_2_-4′β (δ_H_ 1.79)/${\text{H}}_{2}-6^{\prime }$β (δ_H_ 1.14), and H_2_-6′α (δ_H_ 1.87)/H_2_-8′α (δ_H_ 1.31) suggested that ${\text{CH}}_{2}-2^{\prime }$ and ${\text{CH}}_{2}-8^{\prime }$ were on the same face of the ring system, which is different with that of **5**.

Compound **7** was obtained as a yellow oil with a molecular ion peak at *m/z* 559.1938 [M + Na]^+^ in HRESIMS (Figure S62), coinciding with the molecular formula C_30_H_32_O_9_. Comparison of the NMR data (Figures S55–S60) revealed that the structure of **7** was very similar to that of **5**, with the difference being the CH_2_ (6′)-CH (7′) substructure in **5** was replaced by a tri-substituted double bond CH (6′)-C (7′) in **7**, which was deduced from the HMBC correlations from H_2_-8′ (δ_H_ 1.96) to C-6′ (δ_C_ 126.0) and C-7′ (δ_C_ 140.8). The configurations of 6′*Z*, 10′*E*, and 2″*E* in compound **7** were elucidated by ROESY correlations (Figure S61) of H_2_-8′ (δ_H_ 1.96)/H-6′ (δ_H_ 5.43), H-10′ (δ_H_ 5.43)/H_2_-12′ (δ_H_ 4.53), and H-2″ (δ_H_ 6.31)/H-9″ (δ_H_ 7.42).

**Figure 3:**
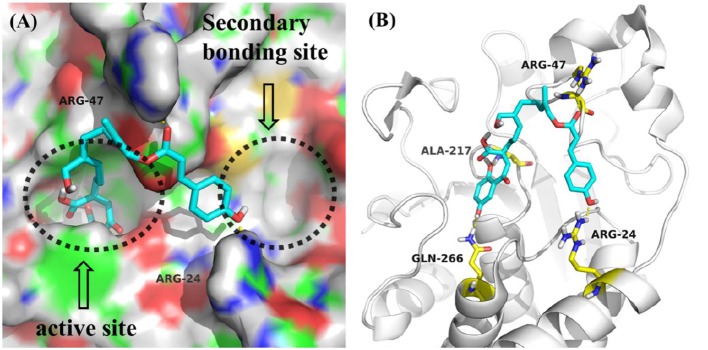
**(A)** Model of compound **1** (cyans) bound to PTP1B (PDB: 1QXK). **(B)** Interactions between compound **1** (cyans) and PTP1B. The key amino acids were shown in yellow.

All compounds isolated were evaluated for their inhibitory activities against PTP1B using *p*NPP as a substrate and cytotoxicities against BEL, K562, SGC7901, A549, and Hela human cell lines using the MTT method. All of the compounds were non-cytotoxic against the tested tumor cell lines. Compounds **1-4** showed obvious inhibitory activity against PTP1B with IC_50_ values of 17, 20, 19, and 23 μM (Figure S64), respectively, comparable to the positive control Na_3_VO_4_ (IC_50_ = 12 μM). A positive effort was made to explain the activity of compound **1** against PTP1B by performing molecular docking (Figure 3). Docking results implied that **1** binds deep in the active site pocket and form H-bonds with ALA-217 and GLN-266, and the 7″-OH also formed H-bond with ARG-24 which was located in the so-called secondary binding site of PTP1B. Thus, it is a potent active molecule against PTP1B, with the ability to interact with both bonding sites. Based on the above research, we believed that it was feasible and reasonable to obtain PTP1B inhibitors with medicinal potential through appropriate structural modifications of these compounds. The cytotoxicity assessment also further provided evidence for this idea, for that all the compounds were inactive against the tested tumor cell lines.

## Conclusions

In summary, seven new meroterpenoids were isolated and identified from the fruiting bodies of *G. ahmadii*. Among them, compounds **1–4** exhibited inhibitory activity against PTP1B but no cytotoxicity against the tested five tumor cell lines, suggesting that it has great potential to obtain new PTP1B inhibitors with medicinal use through appropriate structural modifications of these compounds. The possible mechanisms of these compounds against PTP1B were also revealed by molecular docking experiment. These findings once again proved the great medicinal values of *Ganoderma* fungi.

## Data Availability Statement

All datasets generated for this study are included in the article/Supplementary Material.

## Author Contributions

JG and FK performed the experiments. QM contributed to the bioassays. QX collected the fruiting bodies of *G. ahmadii*. RZ was responsible for edited pictures. YZ and YW designed the work and revised the paper. All authors have approved the final version of the manuscript.

### Conflict of Interest

The authors declare that the research was conducted in the absence of any commercial or financial relationships that could be construed as a potential conflict of interest.
