# Supplementary material for: Meroterpenoids With Protein Tyrosine Phosphatase 1B Inhibitory Activities From the Fruiting Bodies of Ganoderma ahmadii
Source: Front Chem. 2020 Apr 16;8:279. doi: 10.3389/fchem.2020.00279 (PMC7176929; doi:10.3389/fchem.2020.00279)

Supplementary data

Meroterpenoids with Protein Tyrosine Phosphatase 1B Inhibitory Activities from the Fruiting Bodies of *Ganoderma ahmadii*

Jiaocen Guo[a,b,⊥], Fandong Kong[a,⊥], Qingyun Ma[a], Qingyi Xie[a], Renshuai Zhang[c], Haofu Dai[a], Yougen Wu[b,*], and Youxing Zhao[a,*]

[a] Hainan Key Laboratory for Research and Development of Natural Product from Li Folk Medicine, Institute of Tropical Bioscience and Biotechnology, Chinese Academy of Tropical Agriculture Sciences, Haikou 571101, China.

[b] College of Horticulture, Hainan University, Haikou 570228, China.

[c] Qingdao Cancer Institute, the Affiliated Hospital of Qingdao University, Qingdao 266061, China.

[⊥]Jiaocen Guo and Fandong Kong contributed equally to this work.

*Tel/Fax (Y. X. Zhao):+86-898-66989095. E-mail: zhaoyouxing@itbb.org.cn.

Tel/Fax (Y. G. Wu):+86-13637640969. E-mail: wygeng2003@163.com.

Contents

Figure S1. The ^{1}H NMR Spectrum of Compound **1** in CD_3OD 4
Figure S2. The ^{13}C NMR Spectrum of Compound **1** in CD_3OD 4
Figure S3. The DEPT Spectrum of Compound **1** in CD_3OD 5
Figure S4. The HMQC Spectrum of Compound **1** in CD_3OD 5
Figure S5. The HMBC Spectrum of Compound **1** in CD_3OD.......... 6
Figure S6. The COSY Spectrum of Compound **1** in CD_3OD.......... 6
Figure S7. The ROESY Spectrum of Compound **1** in CD_3OD 7
Figure S8. The HRESIMS Spectroscopic Data of Compound **1** 7
Figure S9. The IR Spectrum of Compound **1** 7
Figure S10. The ^{1}H NMR Spectrum of Compound **2** in CD_3OD 8
Figure S11. The ^{13}C NMR Spectrum of Compound **2** in CD_3OD 8
Figure S12. The DEPT Spectrum of Compound **2** in CD_3OD 9
Figure S13. The HMQC Spectrum of Compound **2** in CD_3OD 9
Figure S14. The HMBC Spectrum of Compound **2** in CD_3OD.......... 10
Figure S15. The COSY Spectrum of Compound **2** in CD_3OD.......... 10
Figure S16. The ROESY Spectrum of Compound **2** in CD_3OD 11
Figure S17. The HRESIMS Spectroscopic Data of Compound **2** 11
Figure S18. The IR Spectrum of Compound **2** 11
Figure S19. The ^{1}H NMR Spectrum of Compound **3** in CD_3OD 12
Figure S20. The ^{13}C NMR Spectrum of Compound **3** in CD_3OD 12
Figure S21. The DEPT Spectrum of Compound **3** in CD_3OD 13
Figure S22. The HMQC Spectrum of Compound **3** in CD_3OD 13
Figure S23. The HMBC Spectrum of Compound **3** in CD_3OD.......... 14
Figure S24. The COSY Spectrum of Compound **3** in CD_3OD.......... 14
Figure S25. The ROESY Spectrum of Compound **3** in CD_3OD 15
Figure S26. The HRESIMS Spectroscopic Data of Compound **3** 15
Figure S27. The IR Spectrum of Compound **3** 15
Figure S28. The ^{1}H NMR Spectrum of Compound **4** in CD_3OD 16
Figure S29. The ^{13}C NMR Spectrum of Compound **4** in CD_3OD 16
Figure S30. The DEPT Spectrum of Compound **4** in CD_3OD 17
Figure S31. The HMQC Spectrum of Compound **4** in CD_3OD 17
Figure S32. The HMBC Spectrum of Compound **4** in CD_3OD.......... 18

Figure S33. The COSY Spectrum of Compound **4** in CD_3OD 18
Figure S34. The ROESY Spectrum of Compound **4** in CD_3OD 19
Figure S35. The HRESIMS Spectroscopic Data of Compound **4** 19
Figure S36. The IR Spectrum of Compound **4** 19
Figure S37. The ^1H NMR Spectrum of Compound **5** in CD_3OD 20
Figure S38. The ^{13}C NMR Spectrum of Compound **5** in CD_3OD 20
Figure S39. The DEPT Spectrum of Compound **5** in CD_3OD 21
Figure S40. The HMQC Spectrum of Compound **5** in CD_3OD 21
Figure S41. The HMBC Spectrum of Compound **5** in CD_3OD 22
Figure S42. The COSY Spectrum of Compound **5** in CD_3OD 22
Figure S43. The ROESY Spectrum of Compound **5** in CD_3OD 23
Figure S44. The HRESIMS Spectroscopic Data of Compound **5** 23
Figure S45. The IR Spectrum of Compound **5** 23
Figure S46. The ^1H NMR Spectrum of Compound **6** in CD_3OD 24
Figure S47. The ^{13}C NMR Spectrum of Compound **6** in CD_3OD 24
Figure S48. The DEPT Spectrum of Compound **6** in CD_3OD 25
Figure S49. The HMQC Spectrum of Compound **6** in CD_3OD 25
Figure S50. The HMBC Spectrum of Compound **6** in CD_3OD 26
Figure S51. The COSY Spectrum of Compound **6** in CD_3OD 26
Figure S52. The ROESY Spectrum of Compound **6** in CD_3OD 27
Figure S53. The HRESIMS Spectroscopic Data of Compound **6** 27
Figure S54. The IR Spectrum of Compound **6** 27
Figure S55. The ^1H NMR Spectrum of Compound **7** in CD_3OD 28
Figure S56. The ^{13}C NMR Spectrum of Compound **7** in CD_3OD 28
Figure S57. The DEPT Spectrum of Compound **7** in CD_3OD 29
Figure S58. The HMQC Spectrum of Compound **7** in CD_3OD 29
Figure S59. The HMBC Spectrum of Compound **7** in CD_3OD 30
Figure S60. The COSY Spectrum of Compound **7** in CD_3OD 30
Figure S61. The ROESY Spectrum of Compound **7** in CD_3OD 31
Figure S62. The HRESIMS Spectroscopic Data of Compound **7** 31
Figure S63. The IR Spectrum of Compound 7 31
Figure S64. Inhibition rates of compounds **1-4** against PTP1B 32

Figure S1. The ^{1}H NMR Spectrum of Compound **1** in CD_3OD

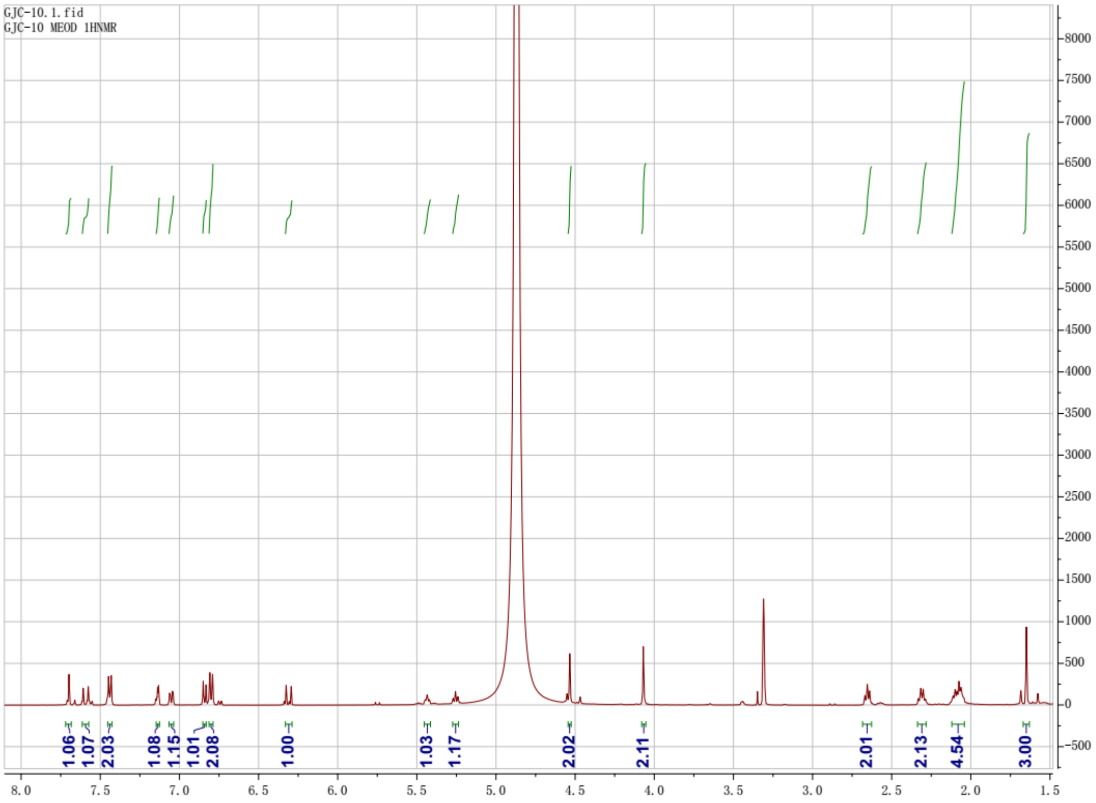

Figure S2. The ^{13}C NMR Spectrum of Compound **1** in CD_3OD

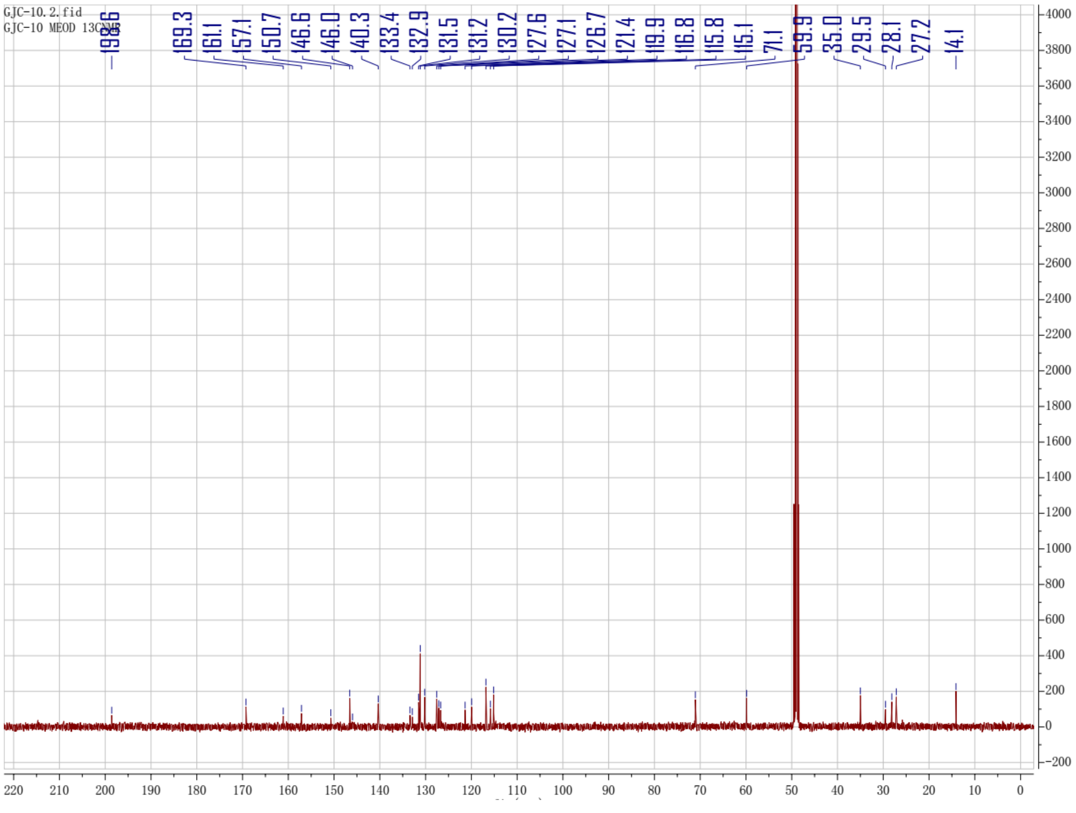

Figure S3. The DEPT Spectrum of Compound **1** in CD_3OD

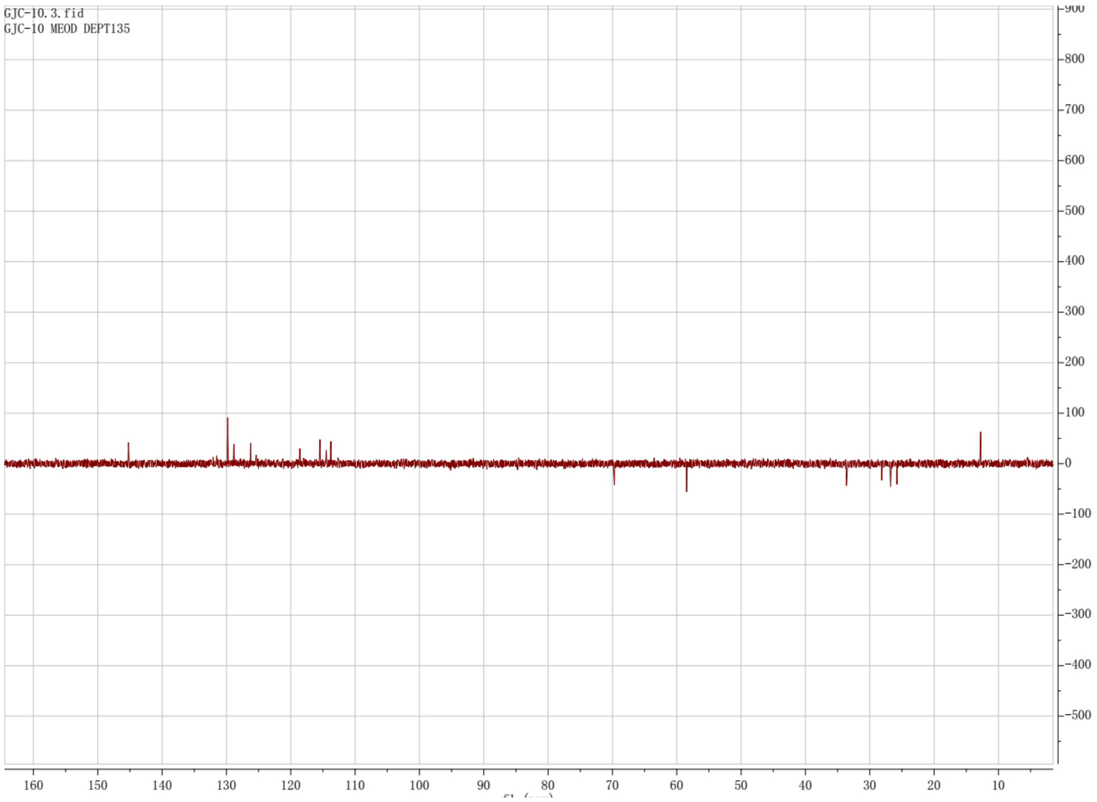

Figure S4. The HMQC Spectrum of Compound **1** in CD_3OD

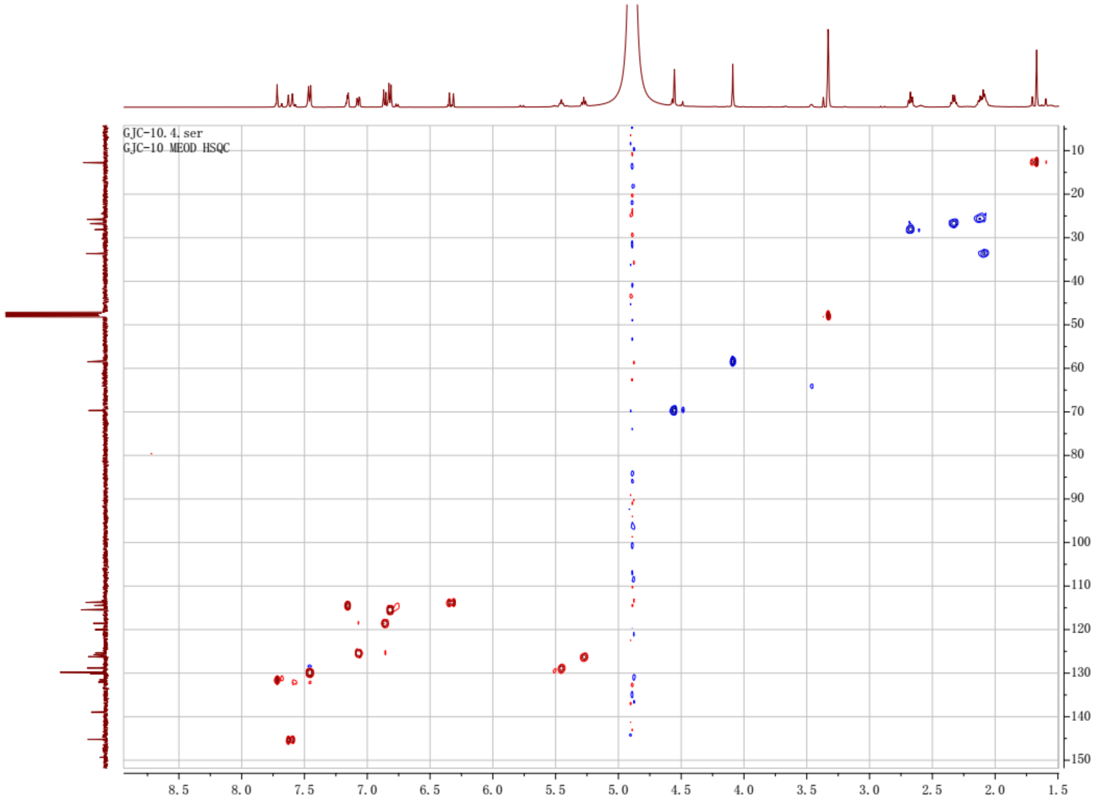

Figure S5. The HMBC Spectrum of Compound **1** in CD_3OD

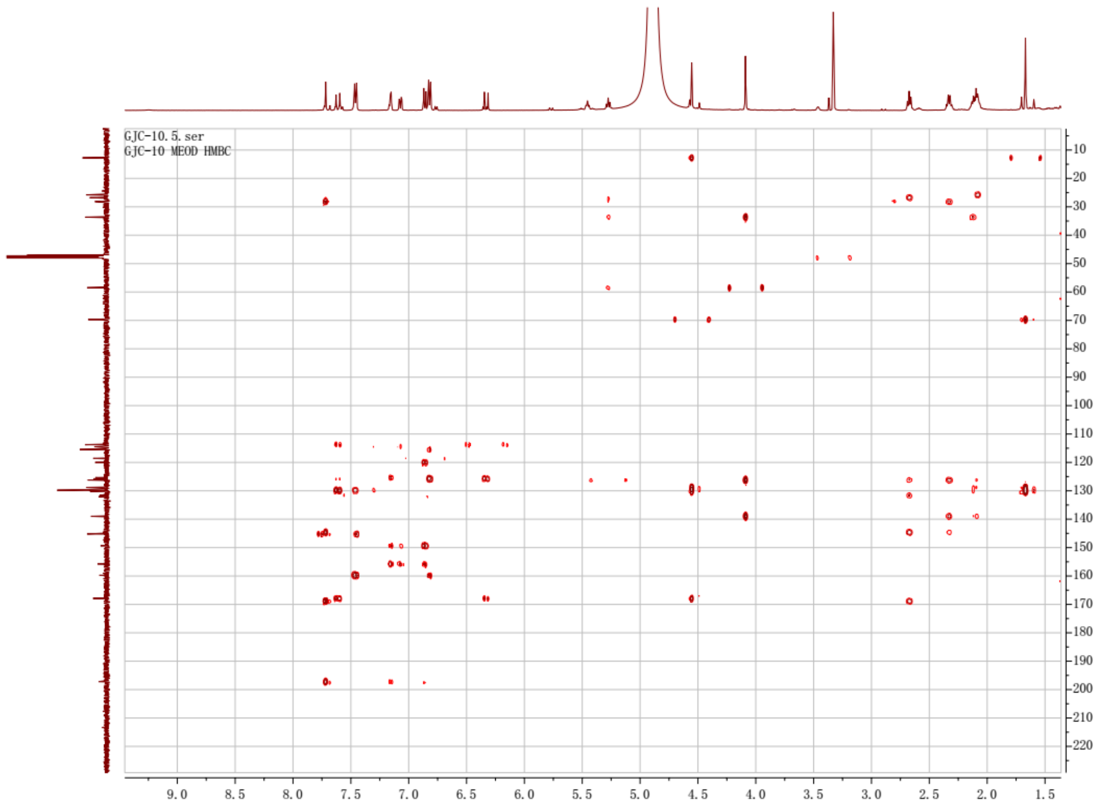

Figure S6. The COSY Spectrum of Compound **1** in CD_3OD

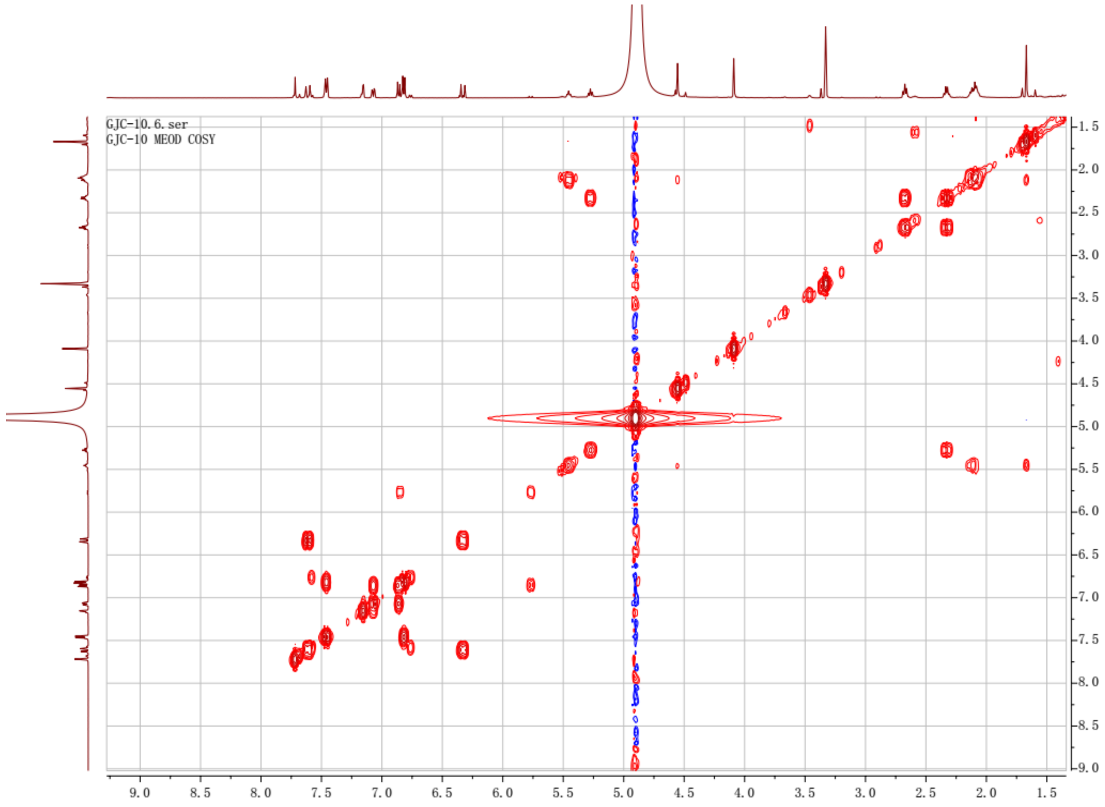

Figure S7. The ROESY Spectrum of Compound **1** in CD_3OD

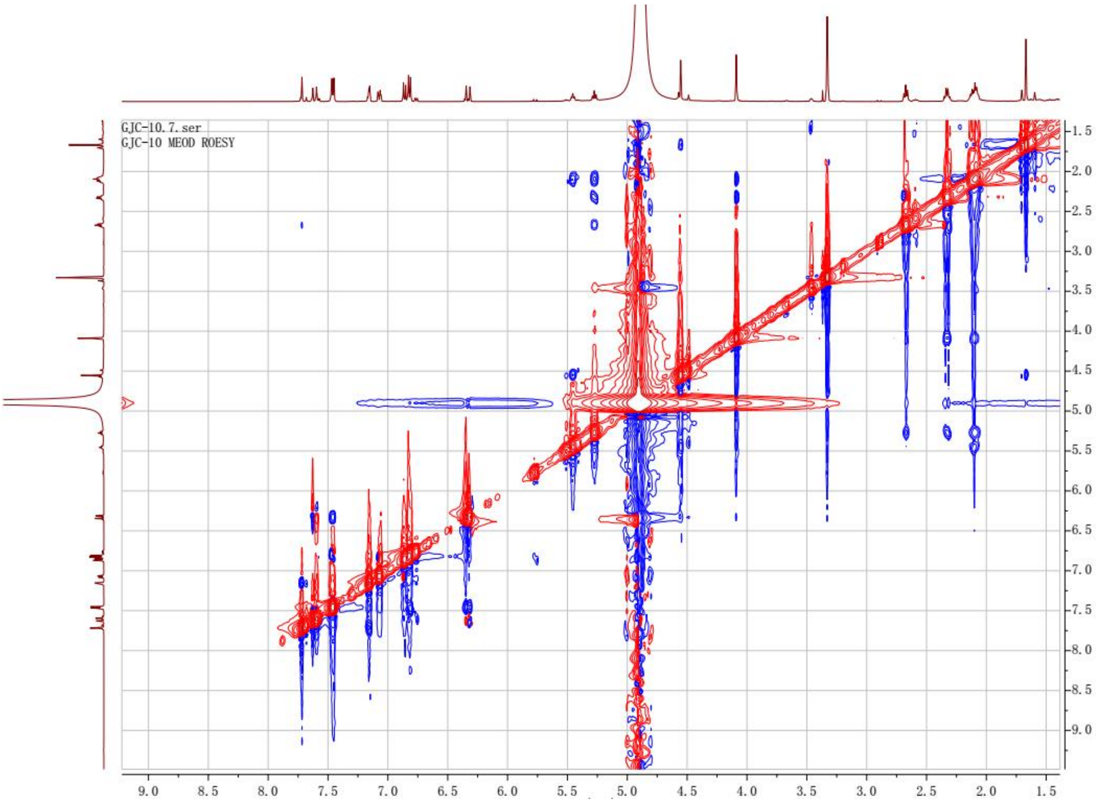

Figure S8. The HRESIMS Spectroscopic Data of Compound **1**

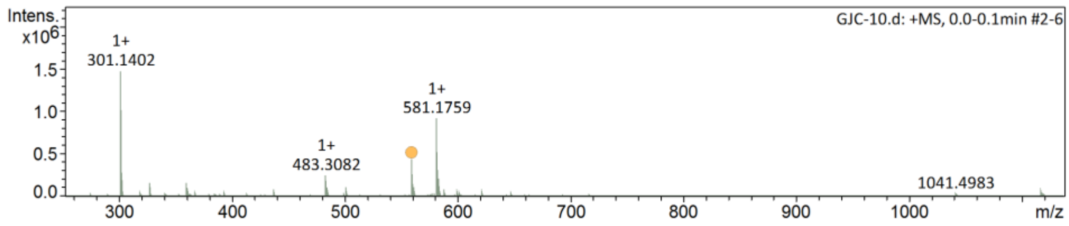

Ion Formula	Meas. m/z	Adduct	err [ppm]	|err| [mDa]
C30H32NaO9	559.193809	M+Na	0.1	0.0

Figure S9. The IR Spectrum of Compound **1**

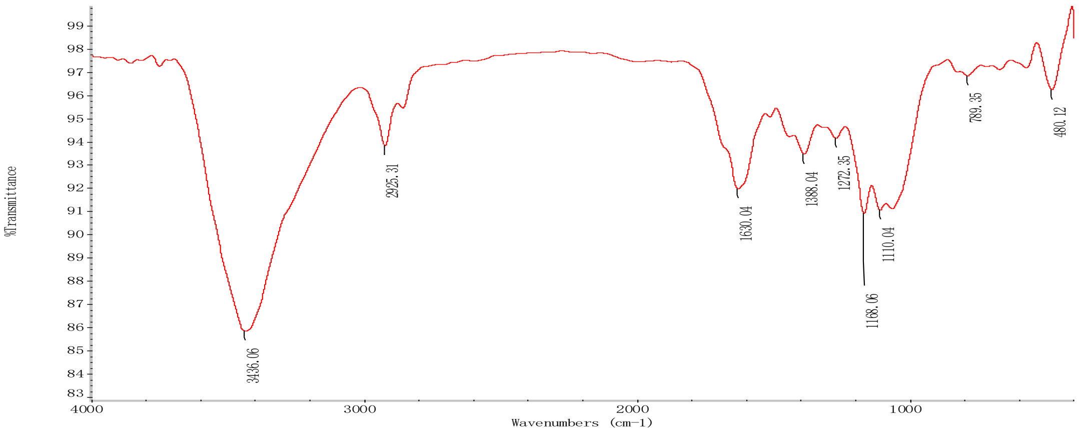

Figure S10. The ^{1}H NMR Spectrum of Compound **2** in CD_3OD

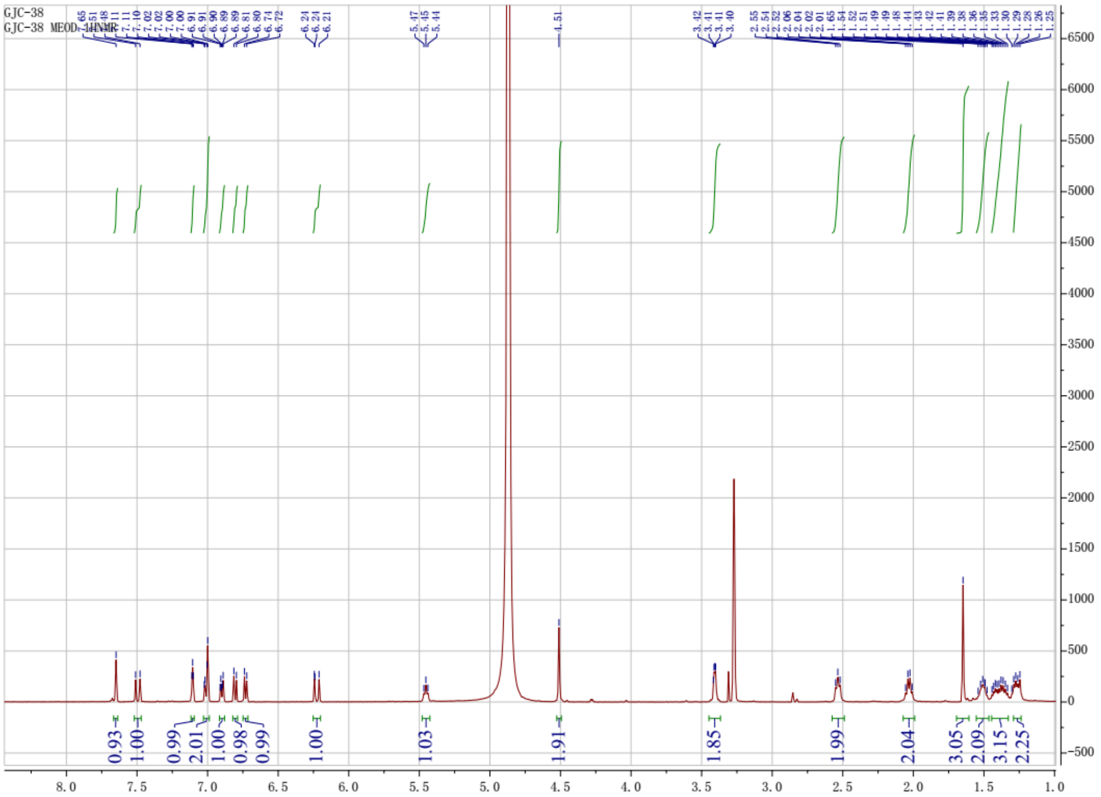

Figure S11. The ^{13}C NMR Spectrum of Compound **2** in CD_3OD

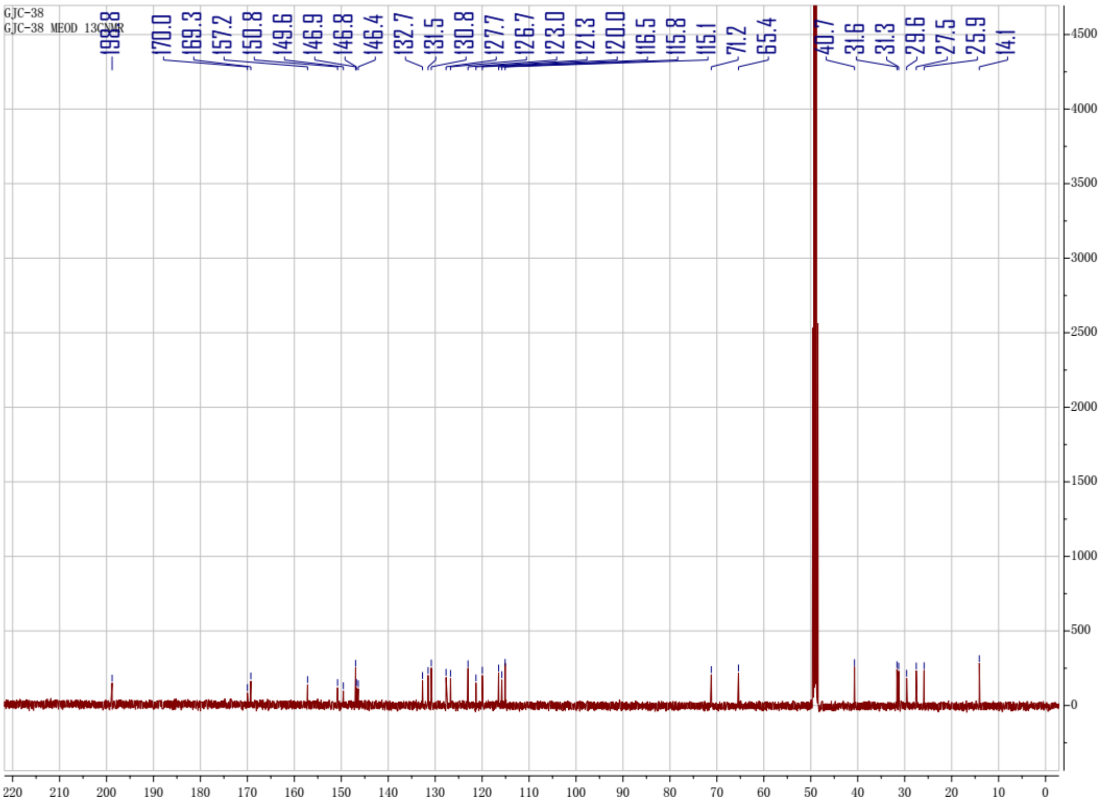

Figure S12. The DEPT Spectrum of Compound **2** in CD_3OD

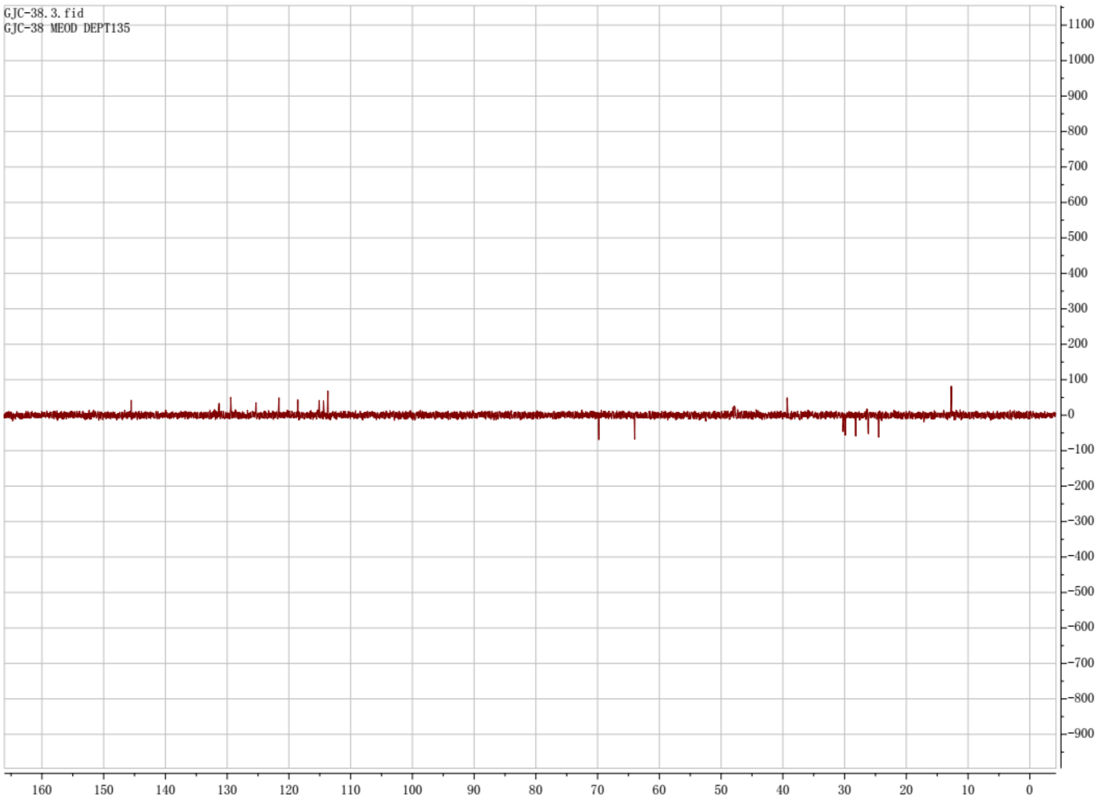

Figure S13. The HMQC Spectrum of Compound **2** in CD_3OD

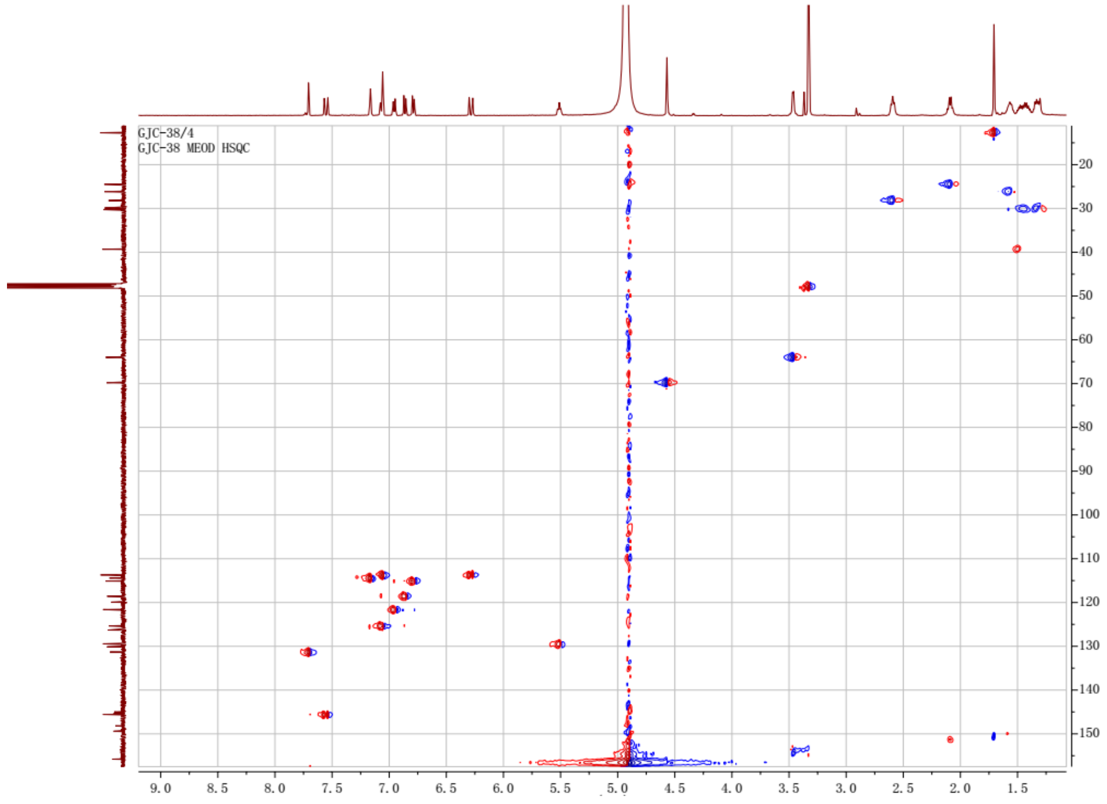

Figure S14. The HMBC Spectrum of Compound **2** in CD_3OD

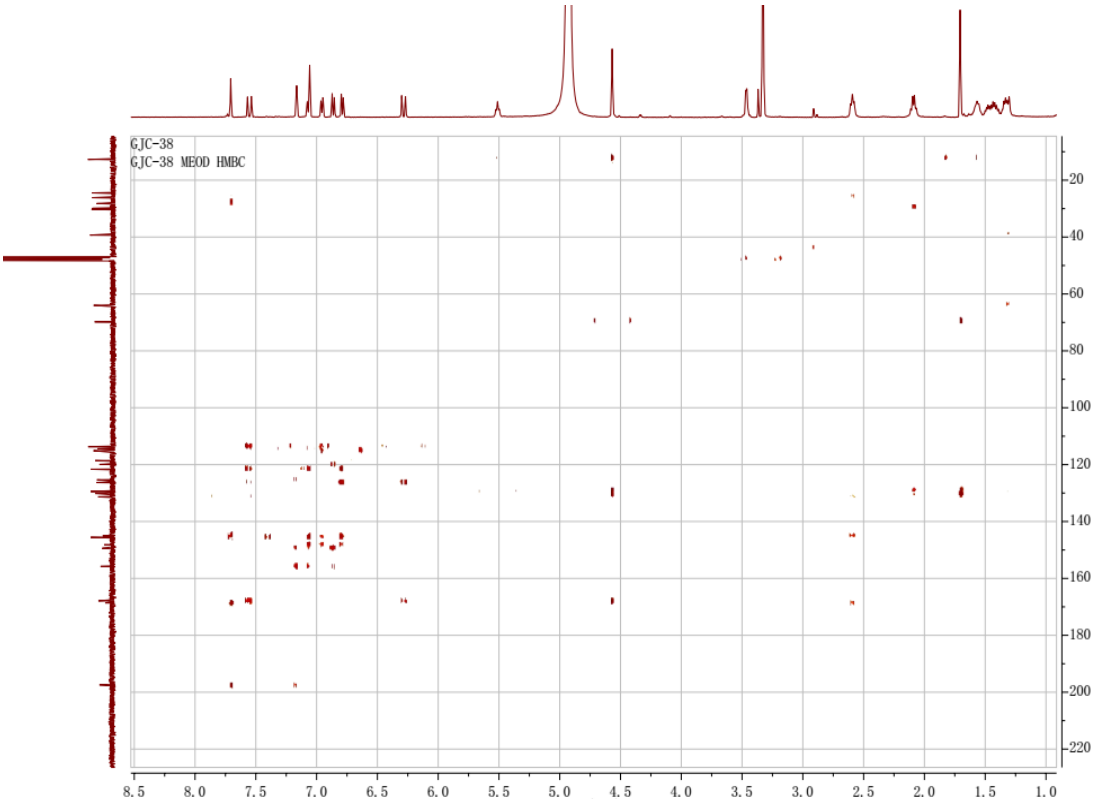

Figure S15. The COSY Spectrum of Compound **2** in CD_3OD

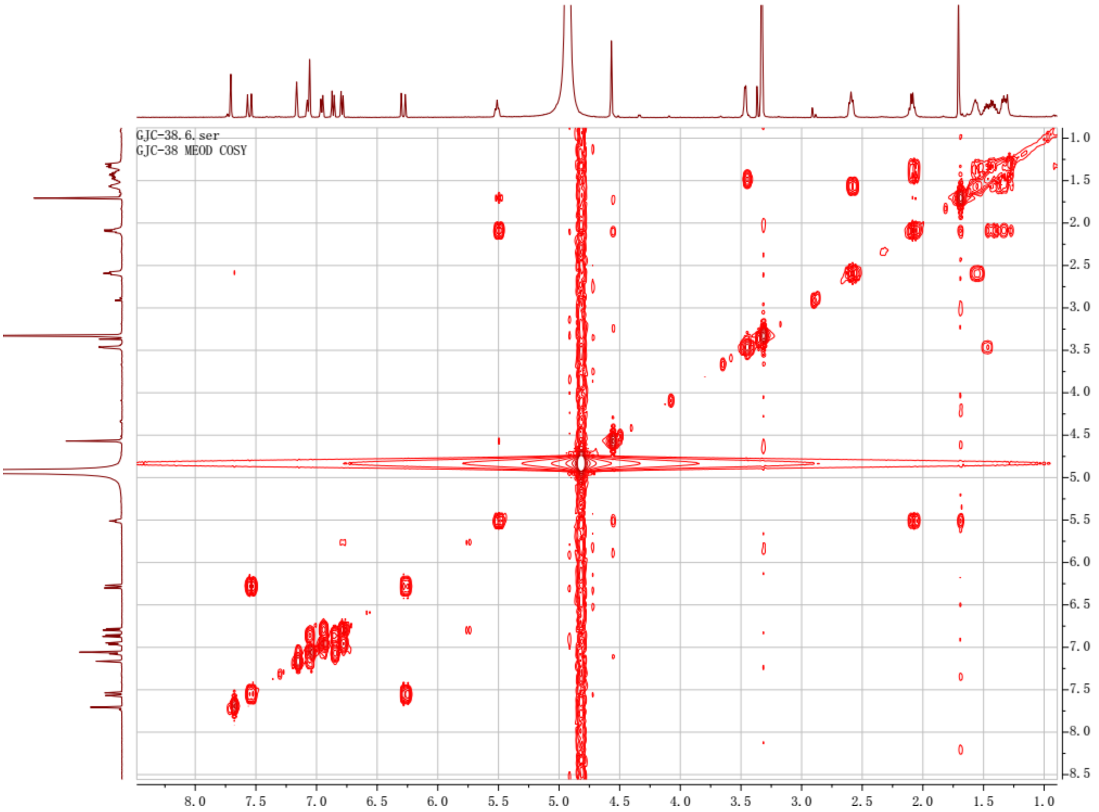

Figure S16. The ROESY Spectrum of Compound **2** in CD_3OD

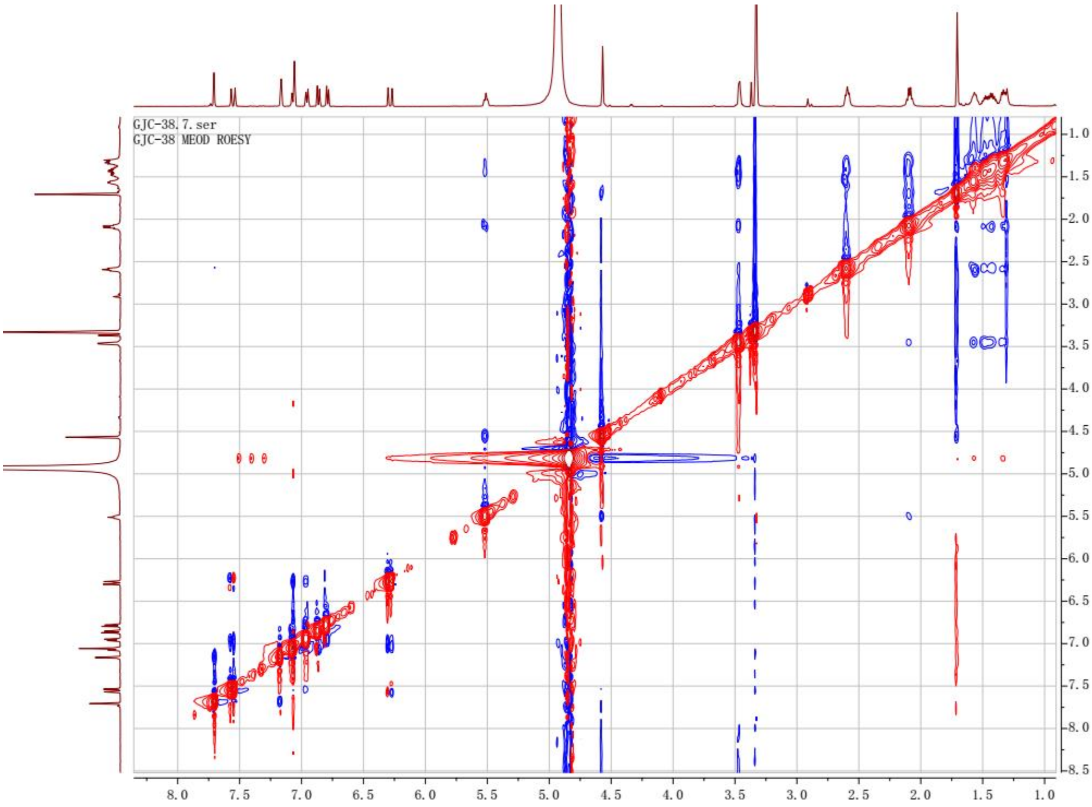

Figure S17. The HRESIMS Spectroscopic Data of Compound **2**

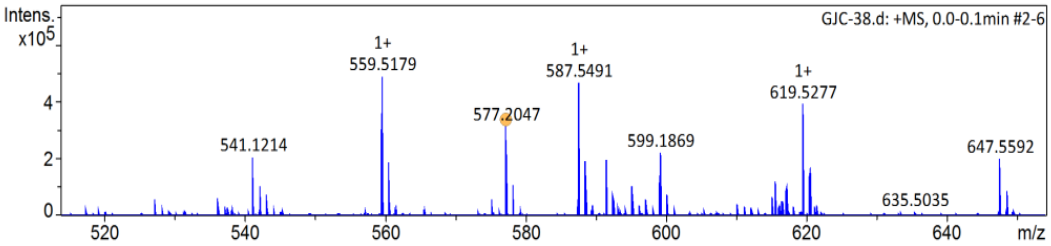

Meas. m/z	#	Ion Formula	m/z	err [ppm]	Mean err [ppm]	rdb	N-Rule	e¯ Conf	mSigma	Adduct
577.204663	1	C30H34NaO10	577.204418	-0.4	-0.8	14.0	ok	even	8.3	M+Na

Figure S18. The IR Spectrum of Compound **2**

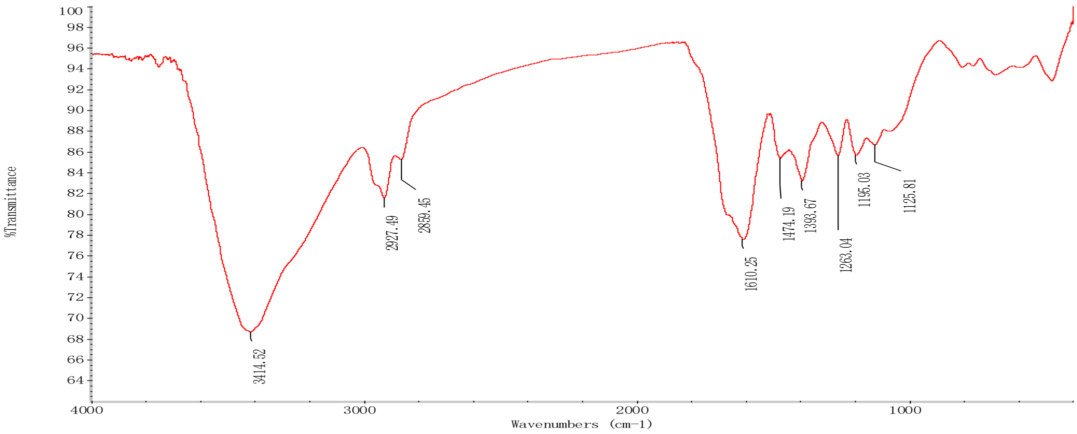

Figure S19. The ^{1}H NMR Spectrum of Compound **3** in CD_3OD

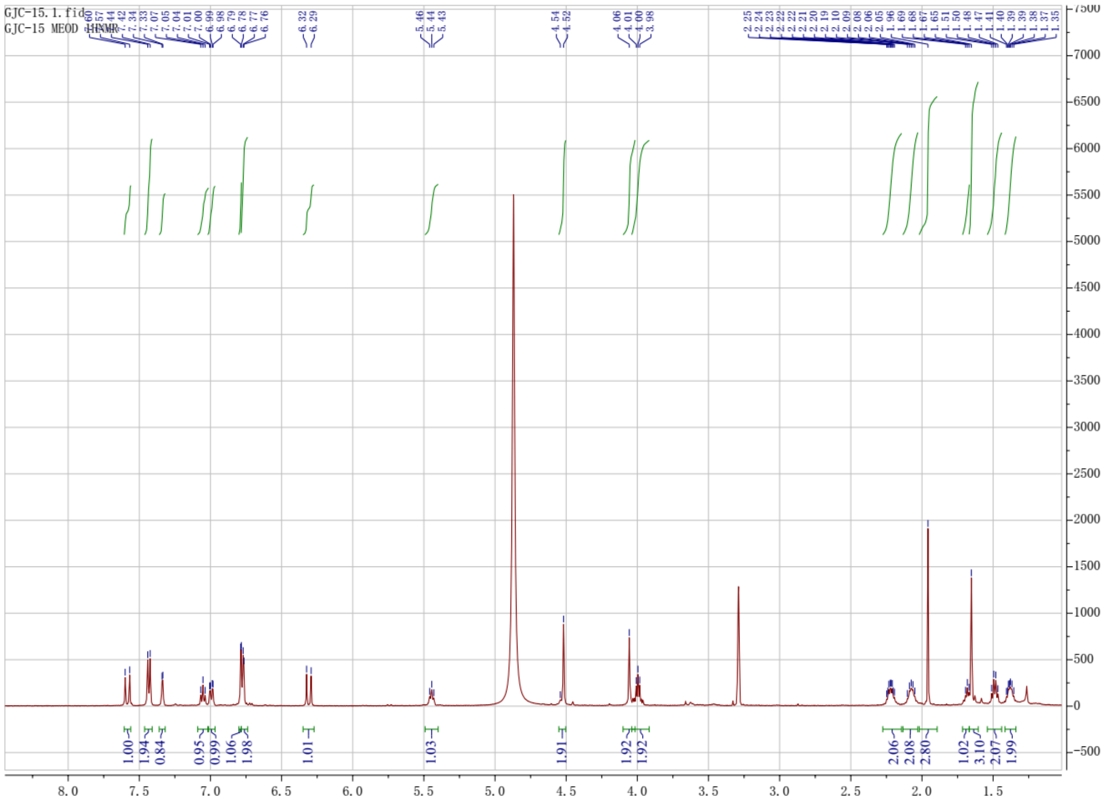

Figure S20. The ^{13}C NMR Spectrum of Compound **3** in CD_3OD

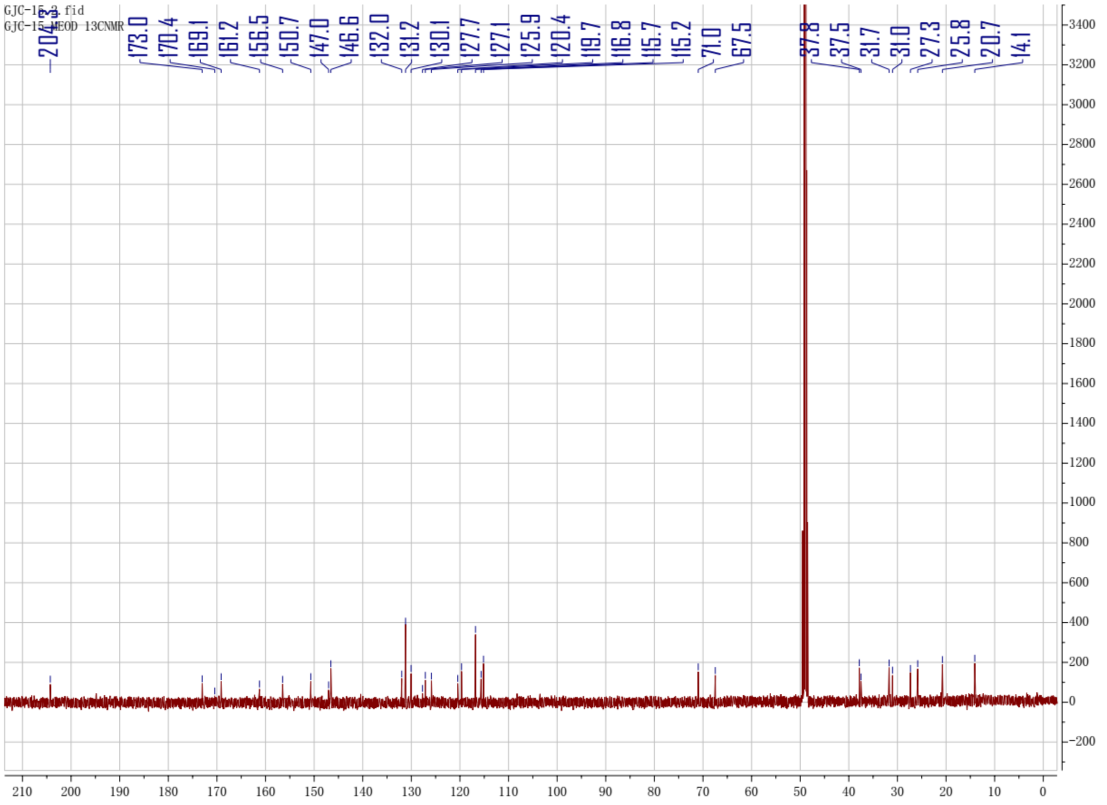

Figure S21. The DEPT Spectrum of Compound **3** in CD_3OD

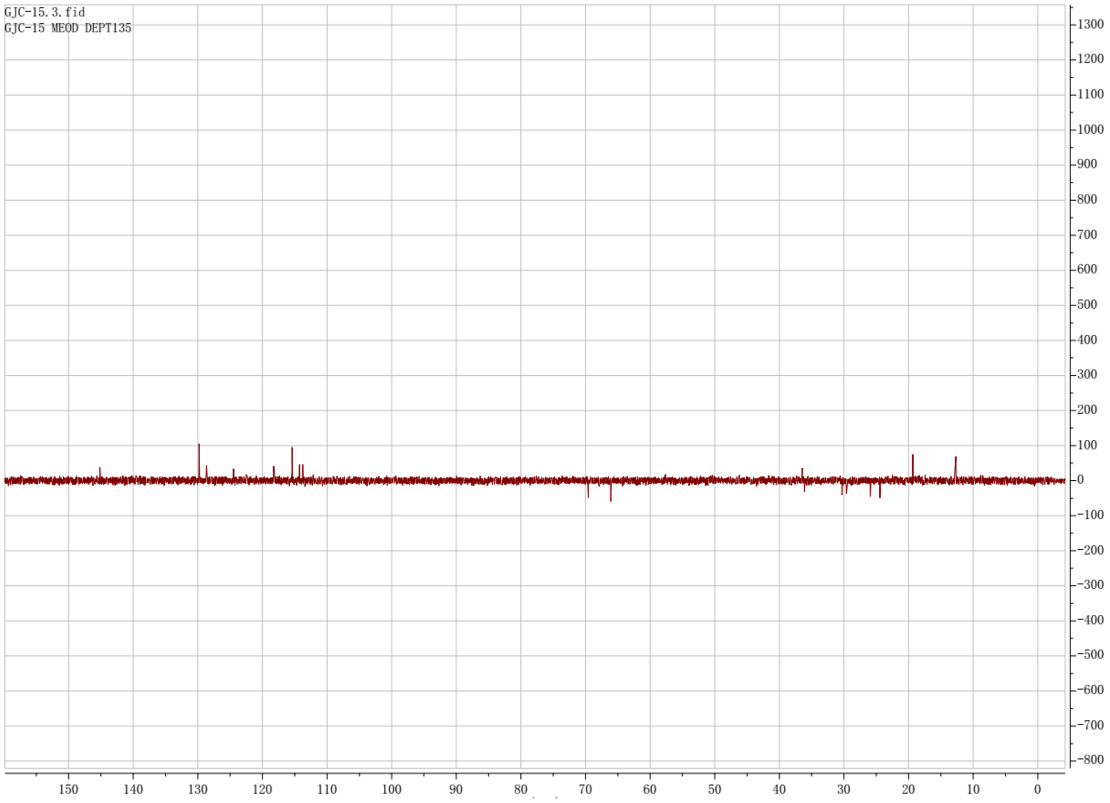

Figure S22. The HMQC Spectrum of Compound **3** in CD_3OD

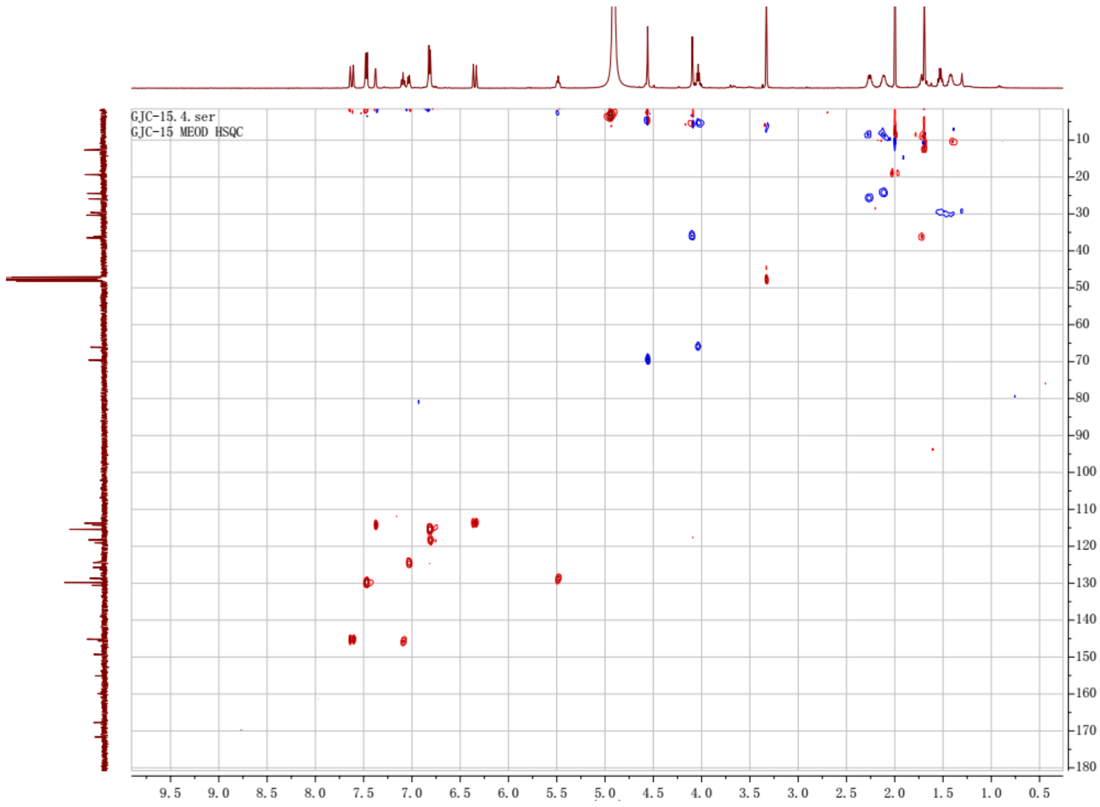

Figure S23. The HMBC Spectrum of Compound **3** in CD_3OD

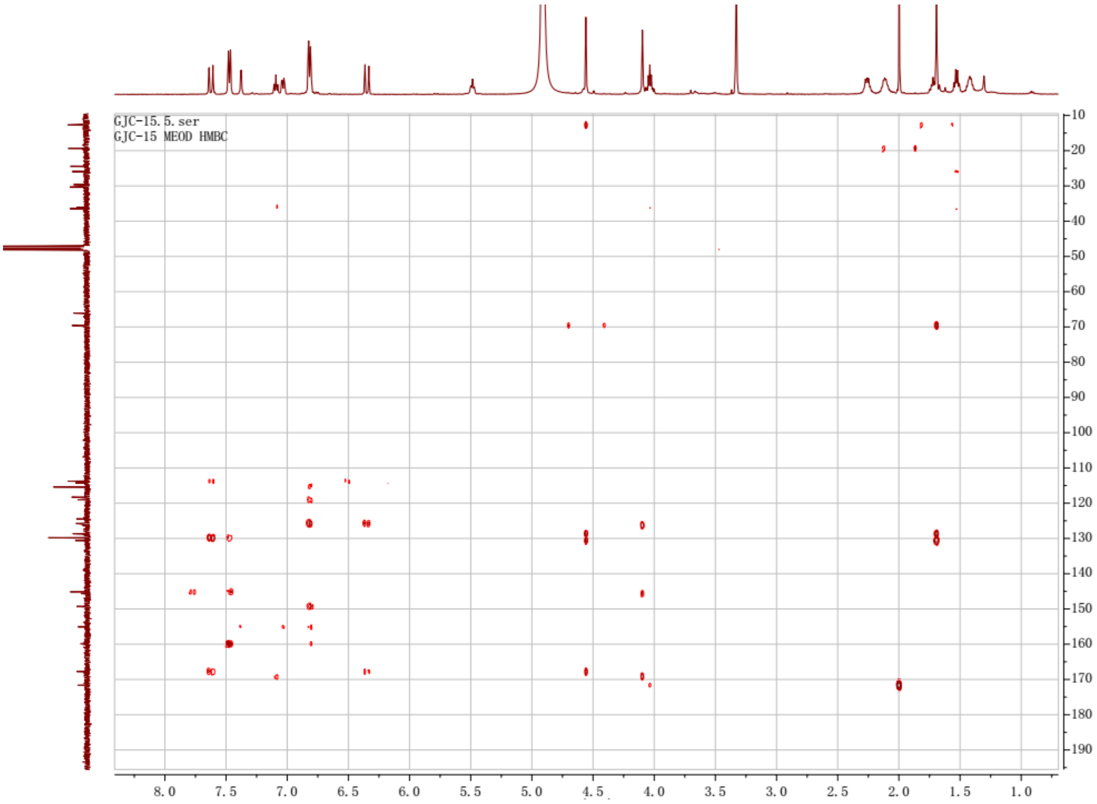

Figure S24. The COSY Spectrum of Compound **3** in CD_3OD

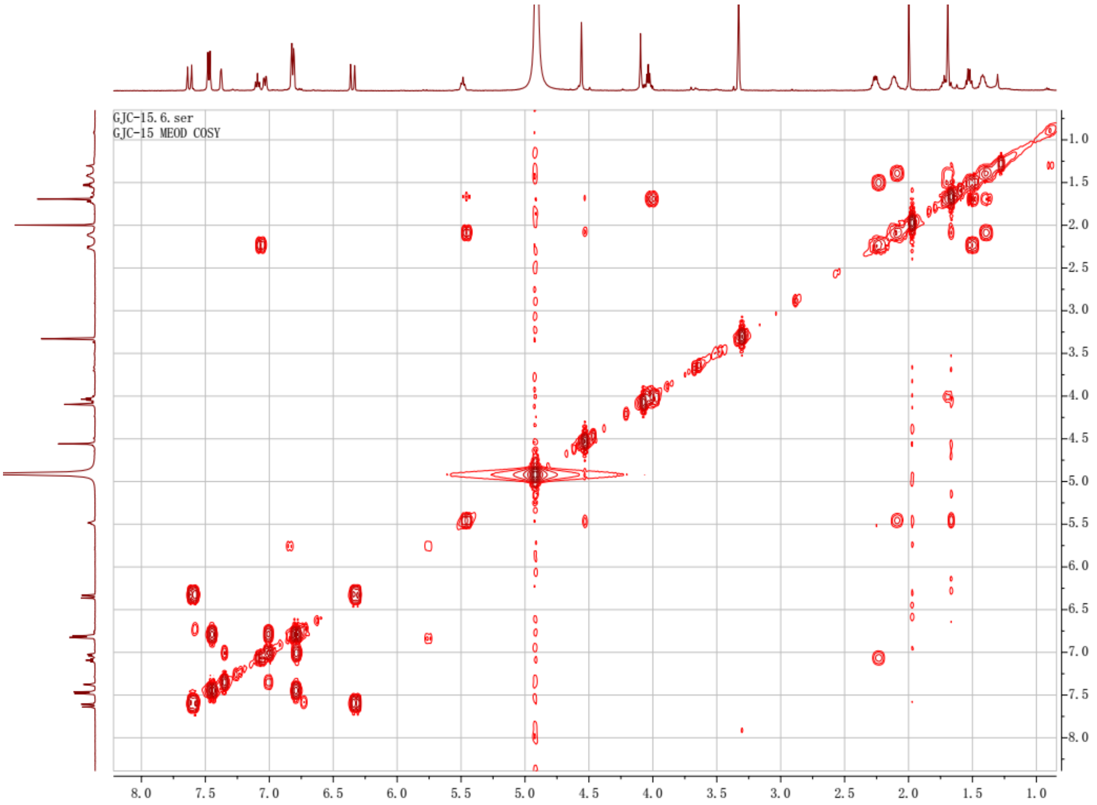

Figure S25. The ROESY Spectrum of Compound **3** in CD_3OD

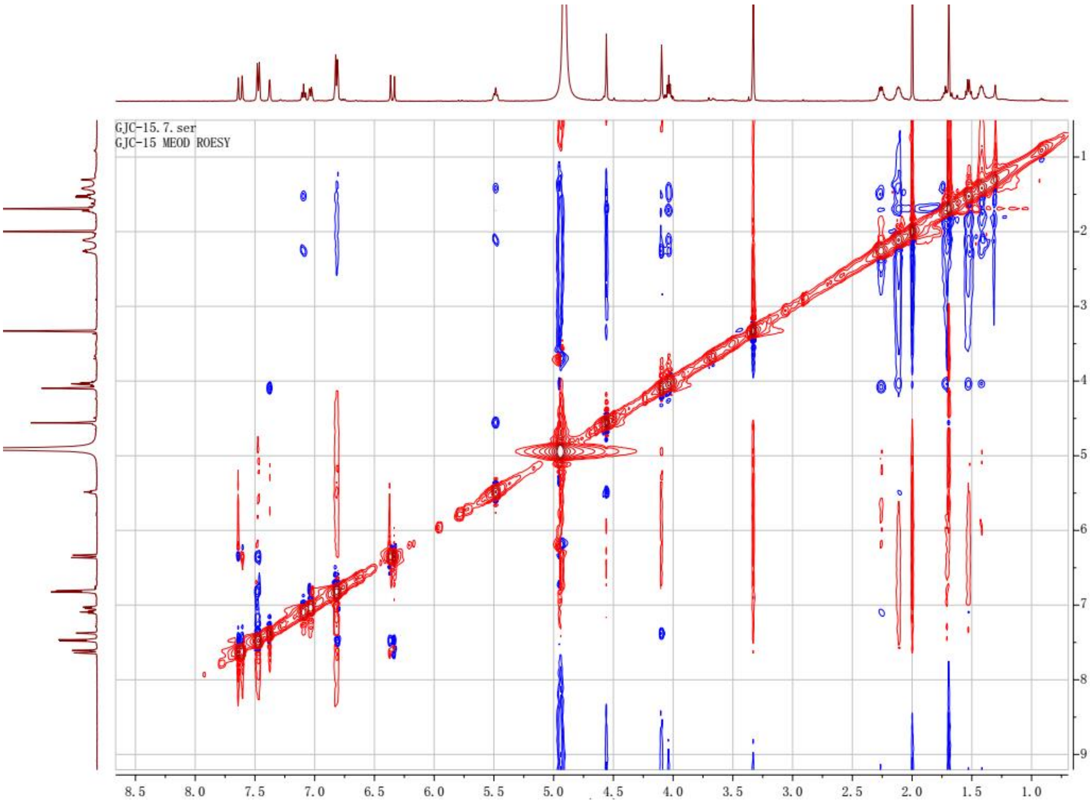

Figure S26. The HRESIMS Spectroscopic Data of Compound **3**

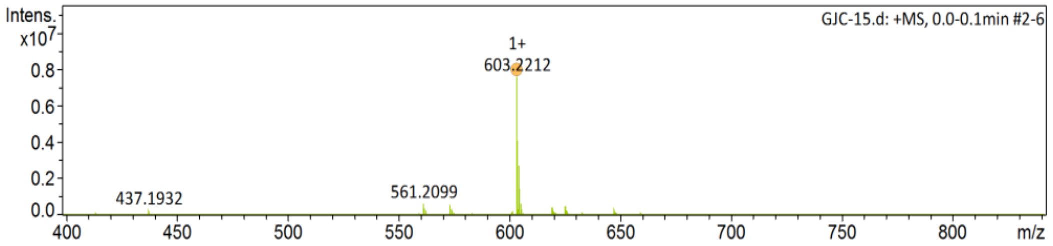

Meas. m/z	#	Ion Formula	m/z	err [ppm]	Mean err [ppm]	rdb	N-Rule	e⁻ Conf	mSigma	Adduct
603.221227	1	C32H36NaO10	603.220068	-1.9	-0.5	15.0	ok	even	1.1	M+Na

Figure S27. The IR Spectrum of Compound **3**

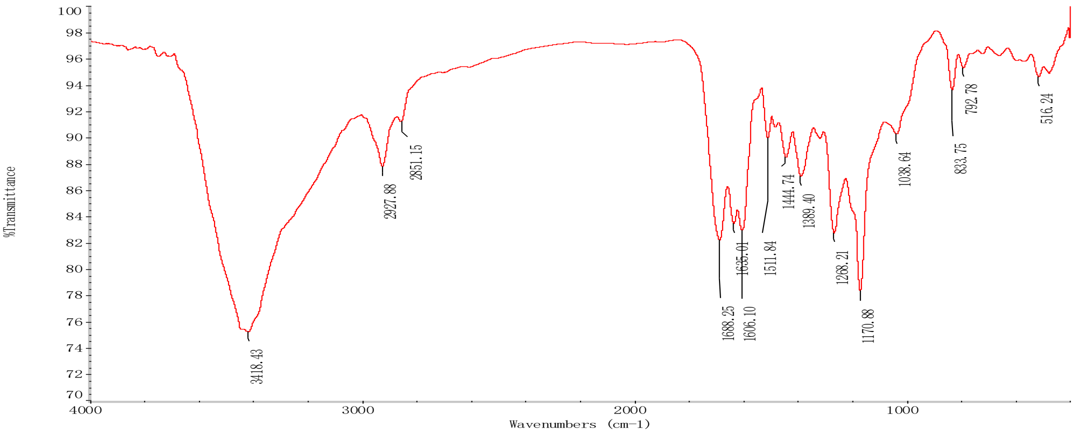

Figure S28. The ^{1}H NMR Spectrum of Compound **4** in CD_3OD

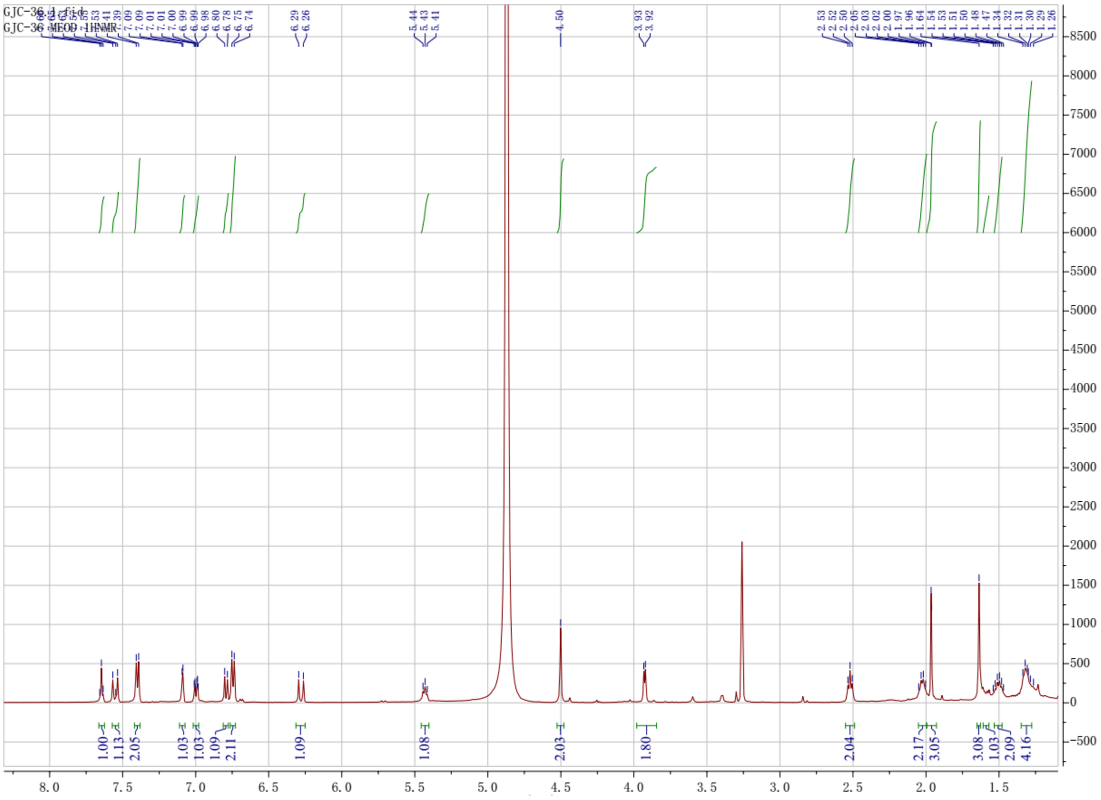

Figure S29. The ^{13}C NMR Spectrum of Compound **4** in CD_3OD

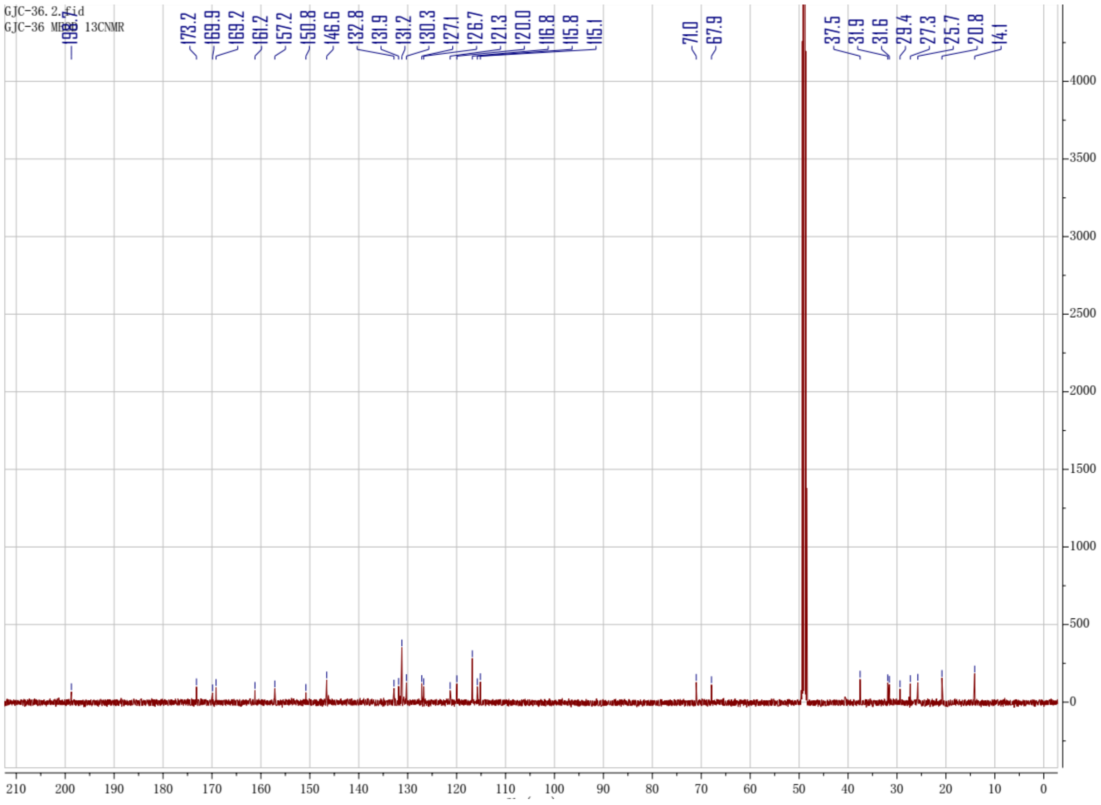

Figure S30. The DEPT Spectrum of Compound **4** in CD_3OD

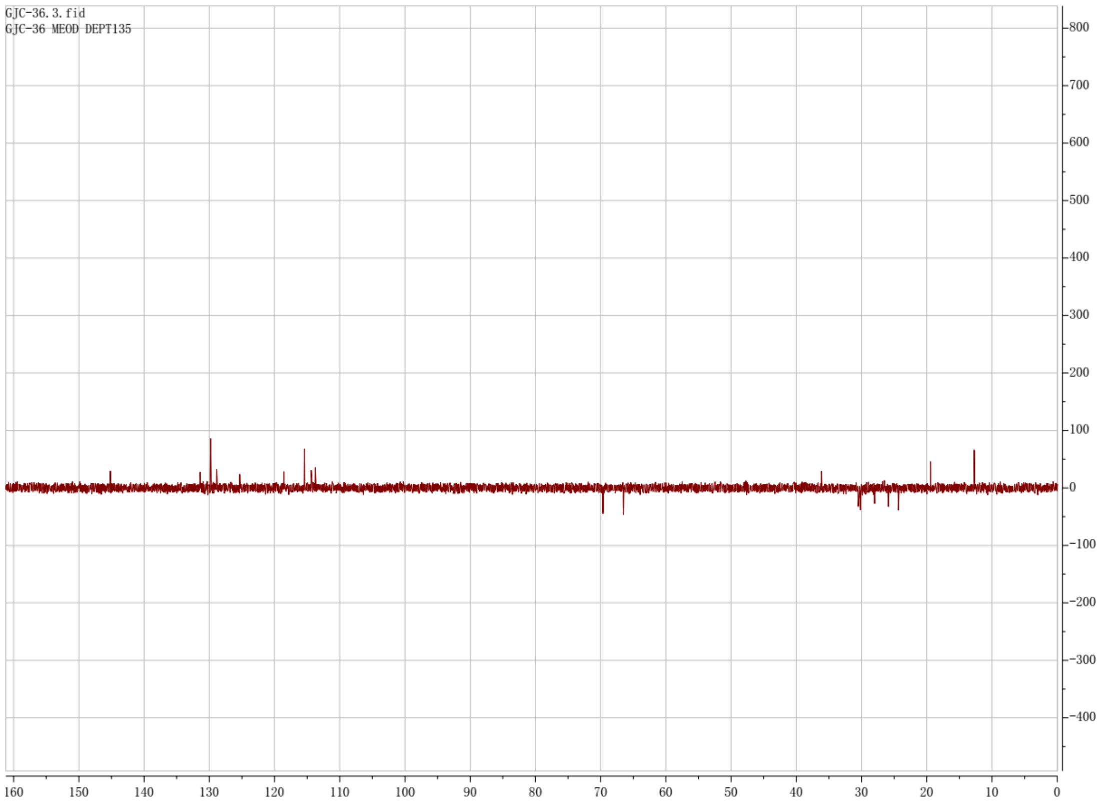

Figure S31. The HMQC Spectrum of Compound **4** in CD_3OD

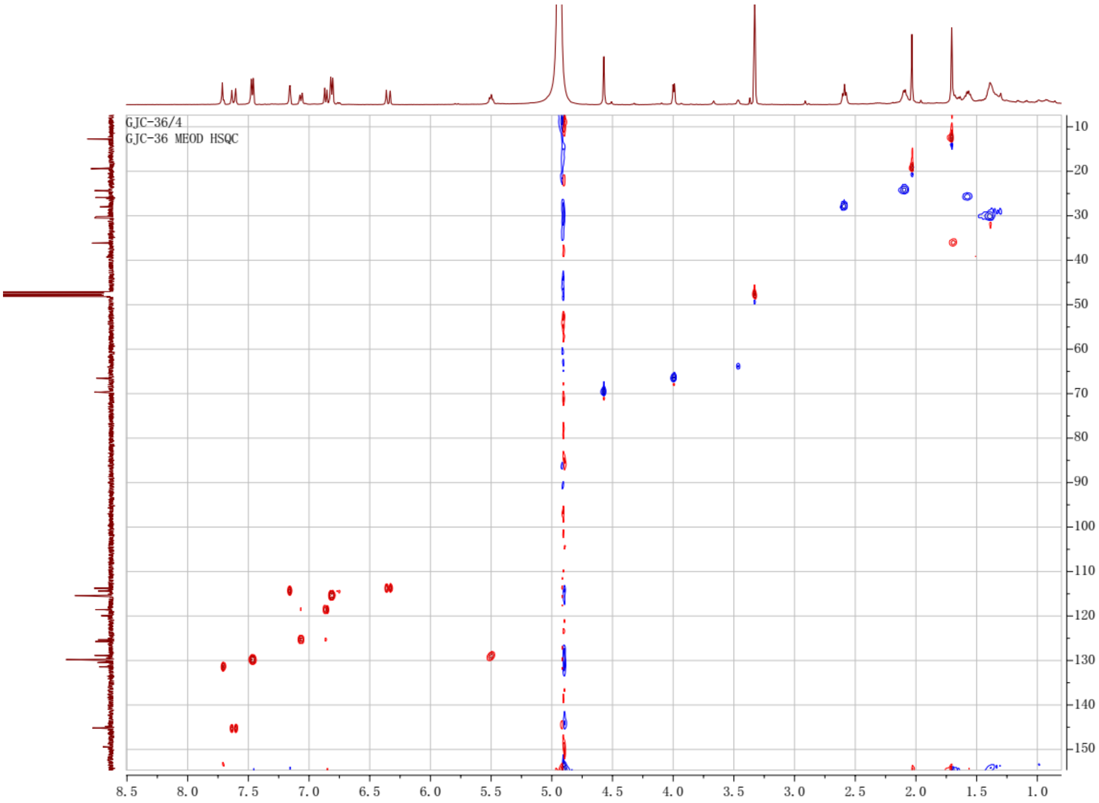

Figure S32. The HMBC Spectrum of Compound **4** in CD_3OD

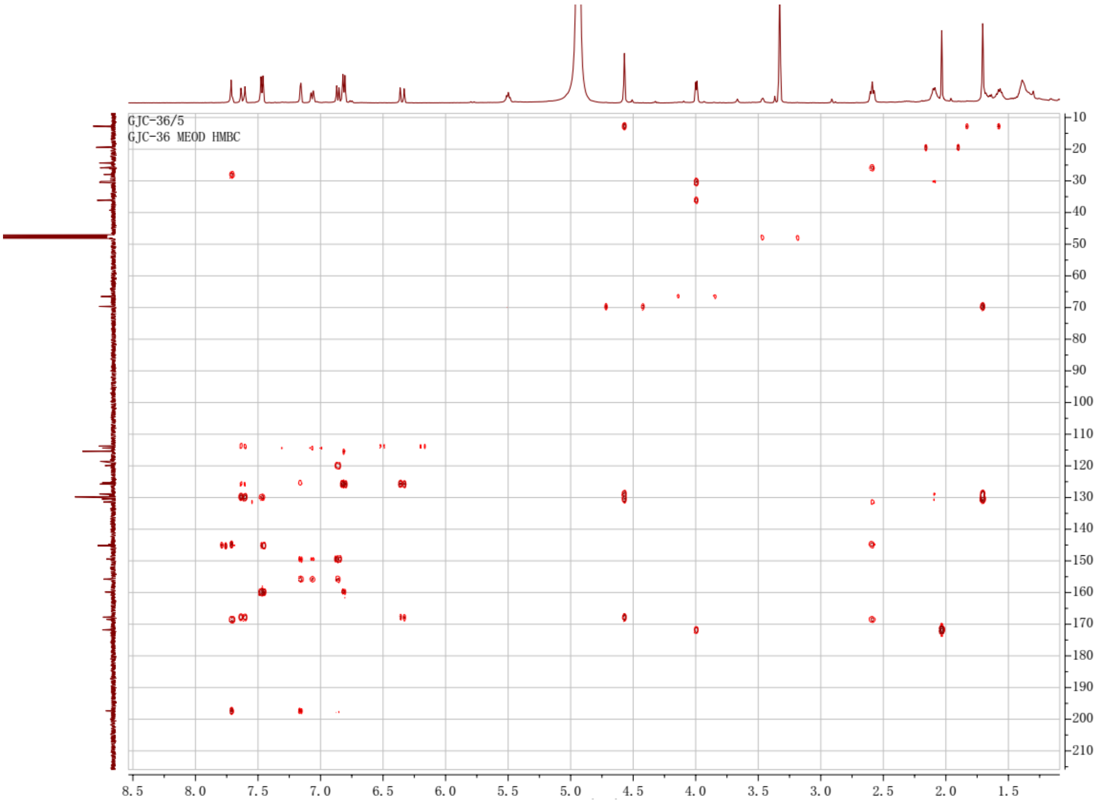

Figure S33. The COSY Spectrum of Compound **4** in CD_3OD

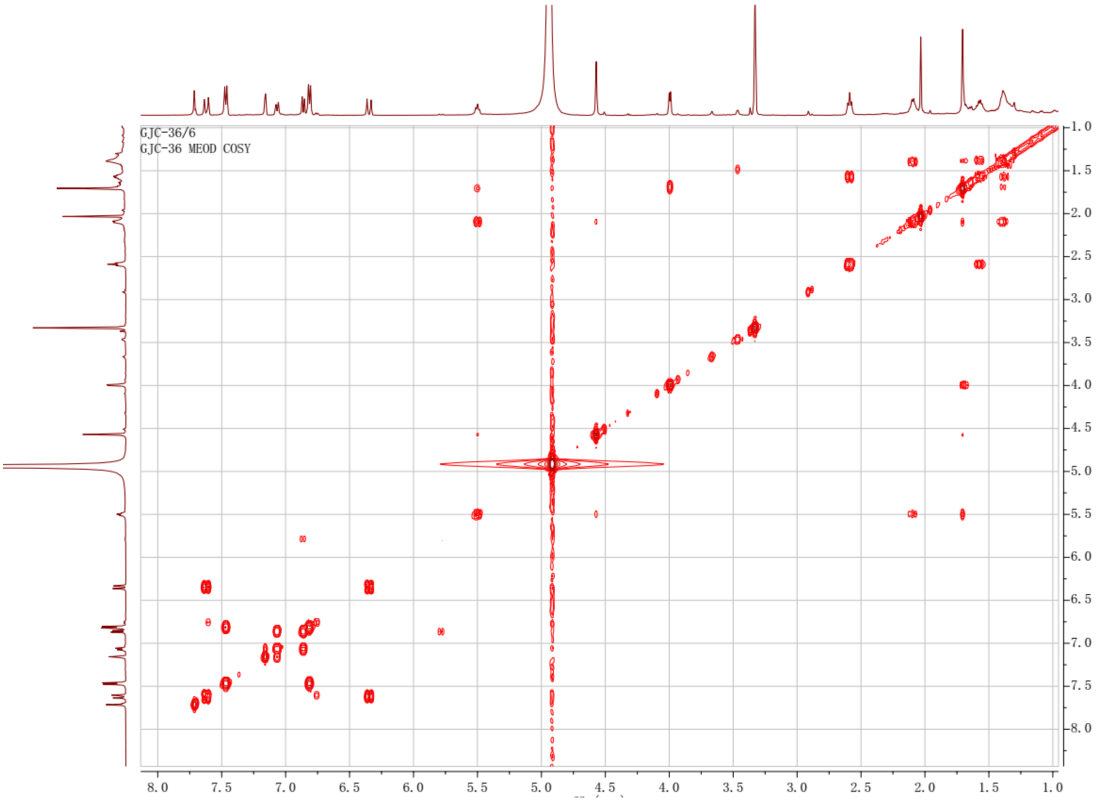

Figure S34. The ROESY Spectrum of Compound **4** in CD_3OD

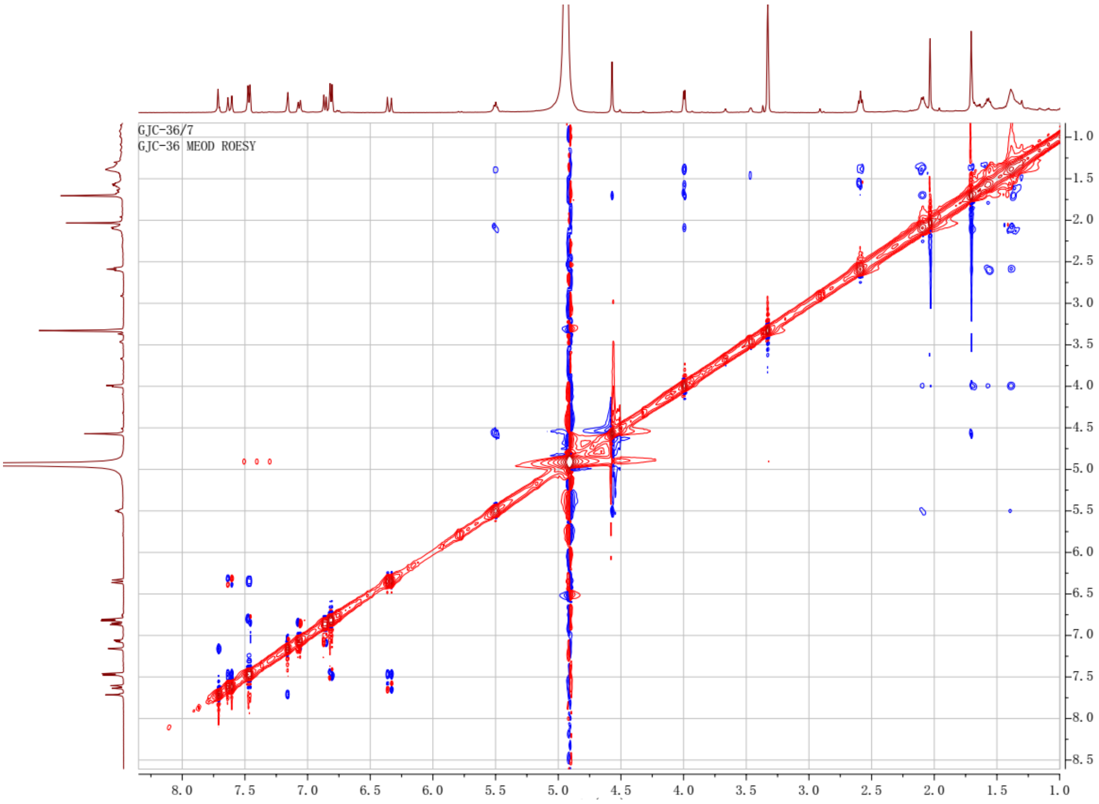

Figure S35. The HRESIMS Spectroscopic Data of Compound **4**

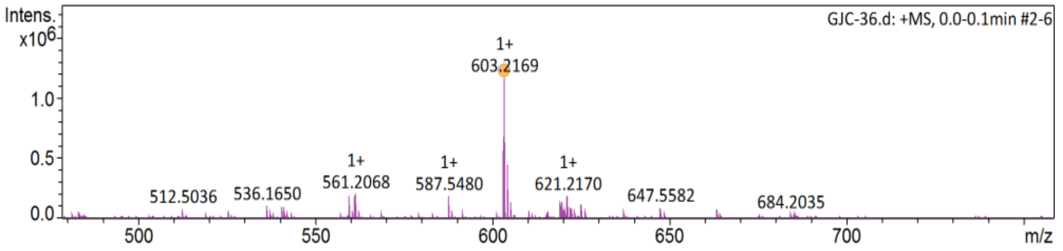

Meas. m/z	#	Ion Formula	m/z	err [ppm]	Mean err [ppm]	rdb	N-Rule	e⁻ Conf	mSigma	Adduct
603.216916	1	C32H36NaO10	603.220068	5.2	8.6	15.0	ok	even	23.1	M+Na

Figure S36. The IR Spectrum of Compound **4**

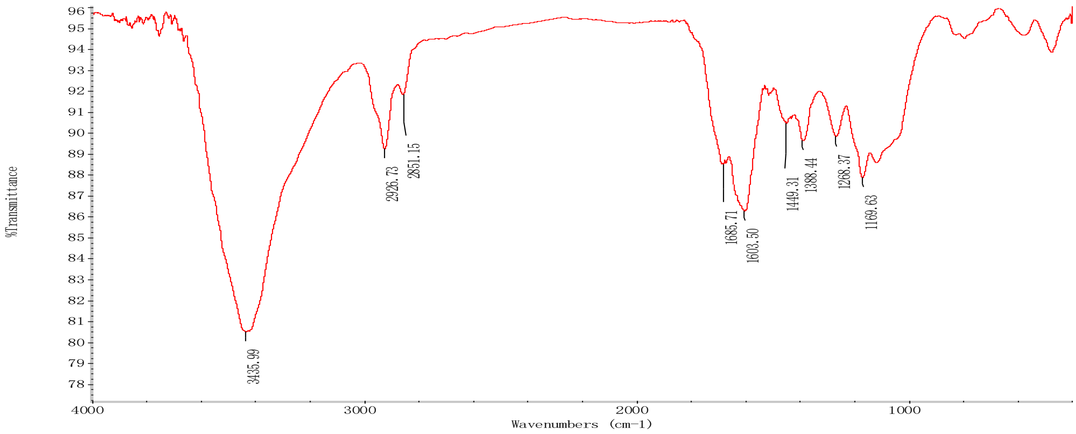

Figure S37. The ^{1}H NMR Spectrum of Compound **5** in CD_3OD

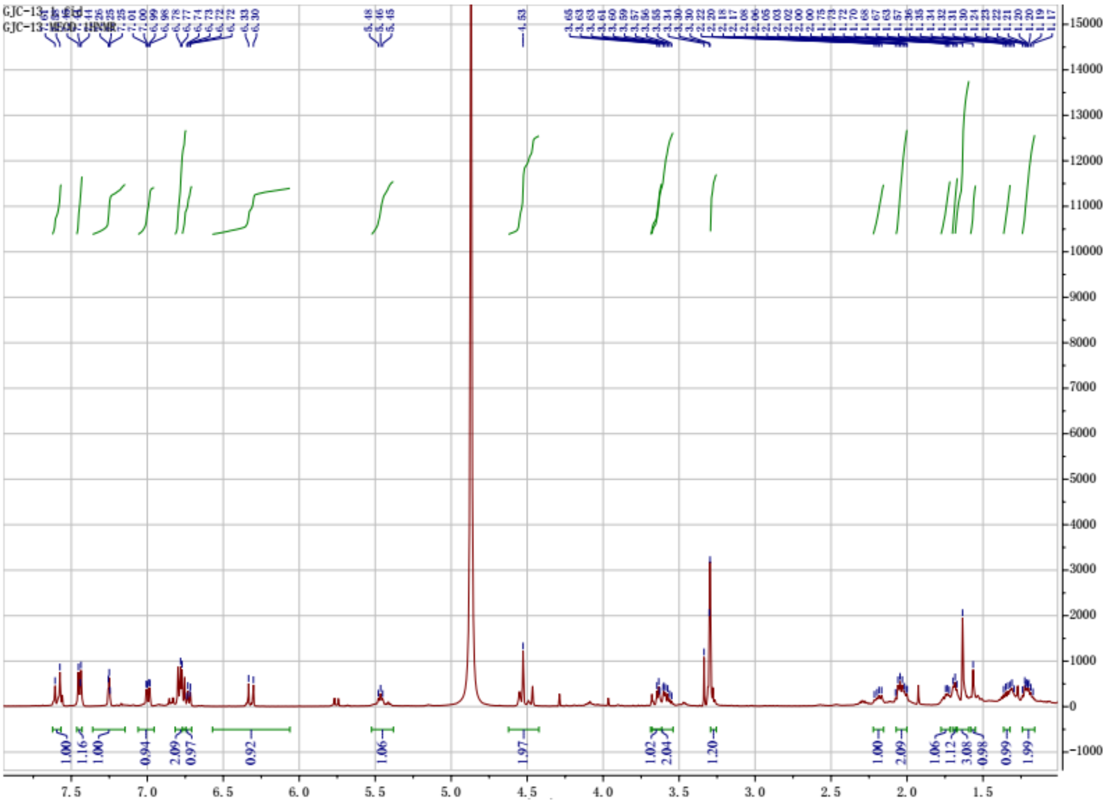

Figure S38. The ^{13}C NMR Spectrum of Compound **5** in CD_3OD

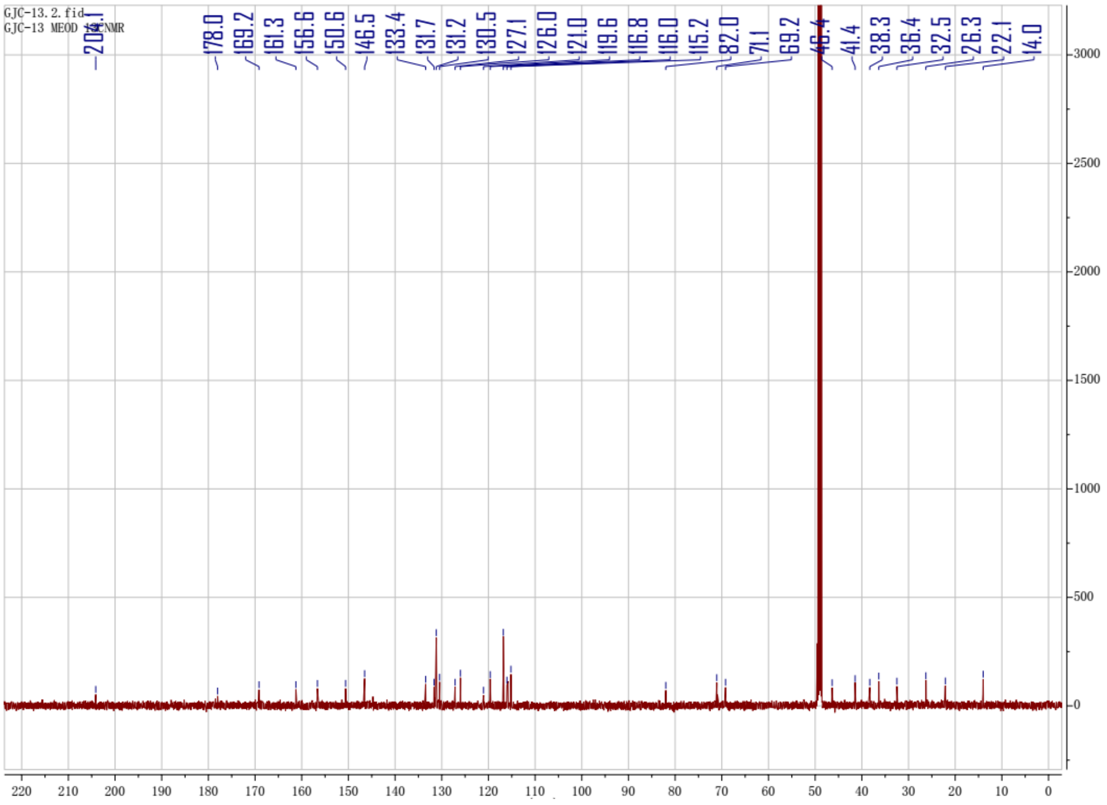

Figure S39. The DEPT Spectrum of Compound **5** in CD_3OD

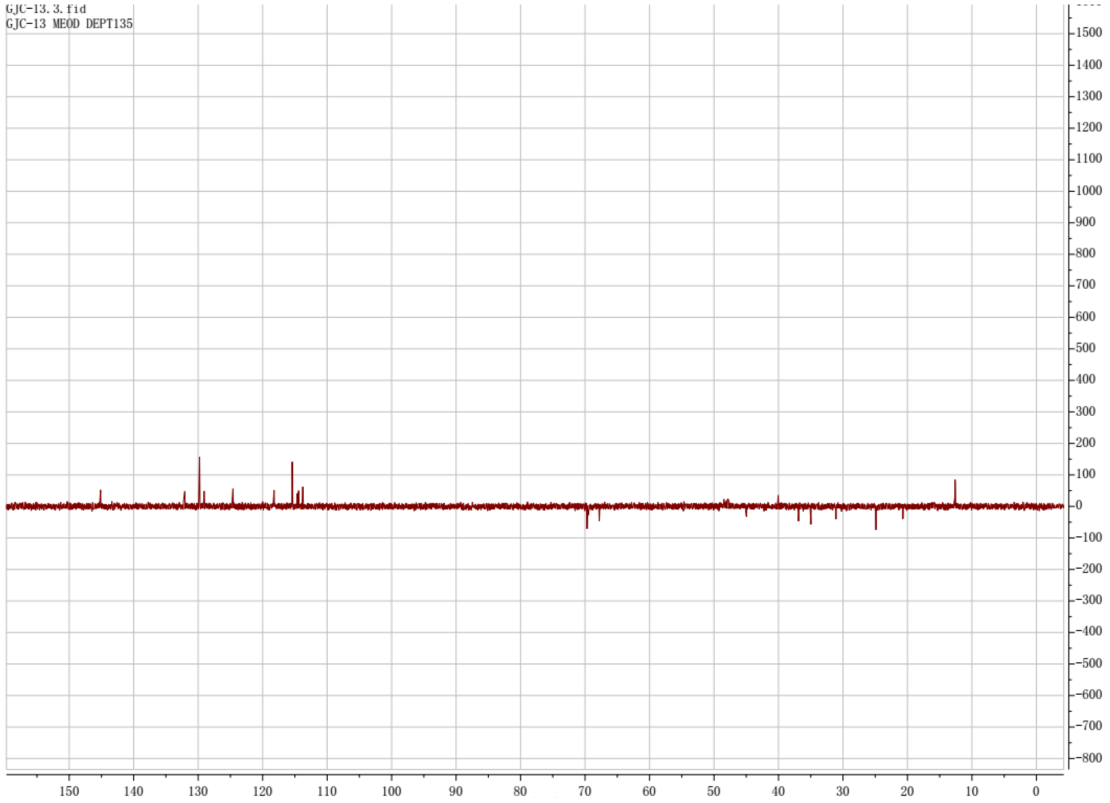

Figure S40. The HMQC Spectrum of Compound **5** in CD_3OD

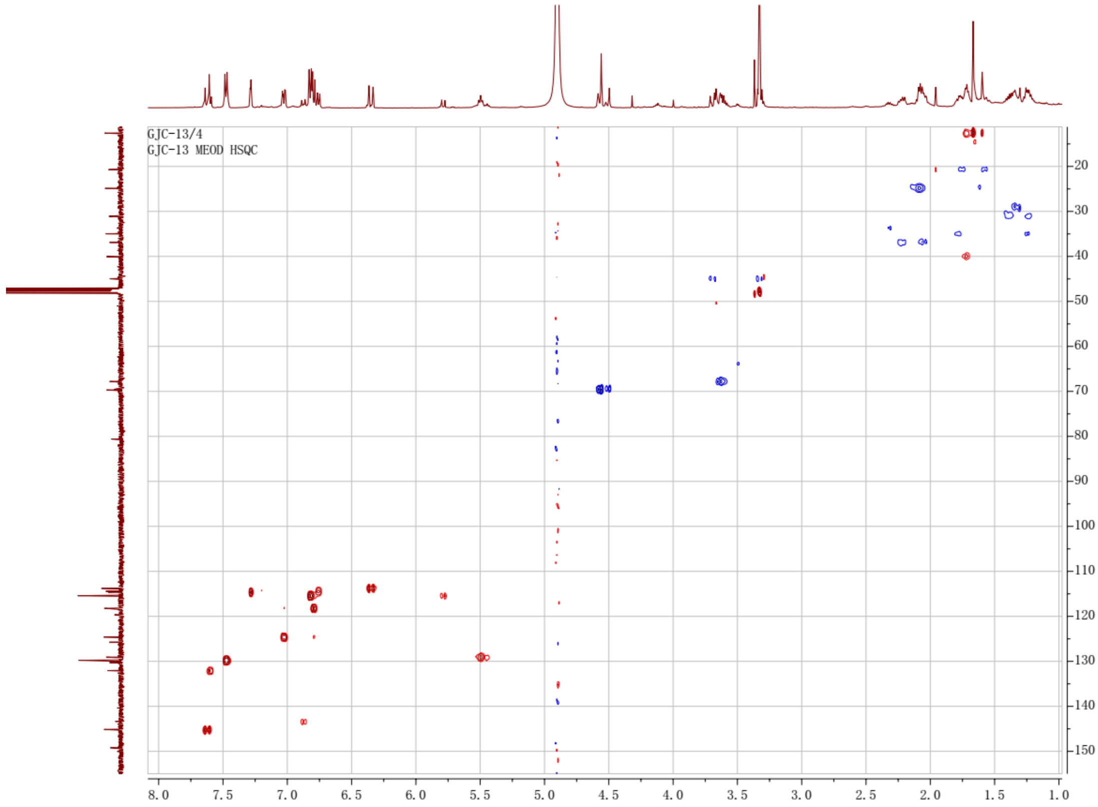

Figure S41. The HMBC Spectrum of Compound **5** in CD_3OD

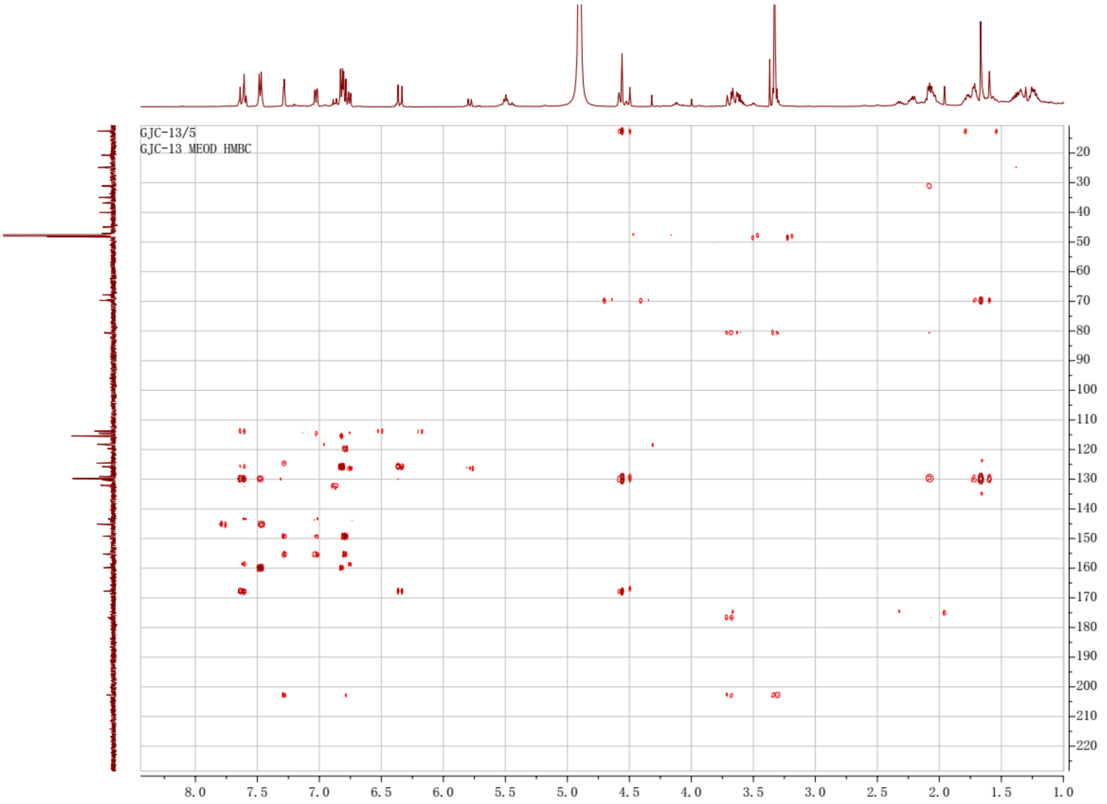

Figure S42. The COSY Spectrum of Compound **5** in CD_3OD

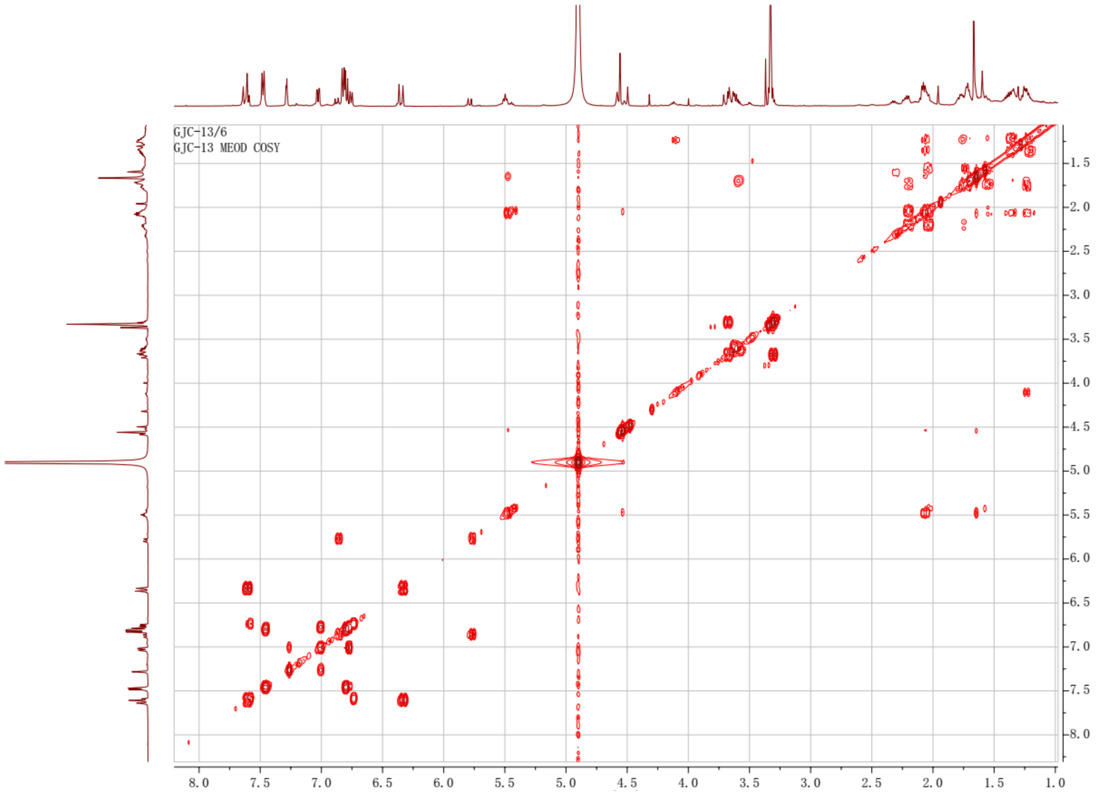

Figure S43. The ROESY Spectrum of Compound **5** in CD_3OD

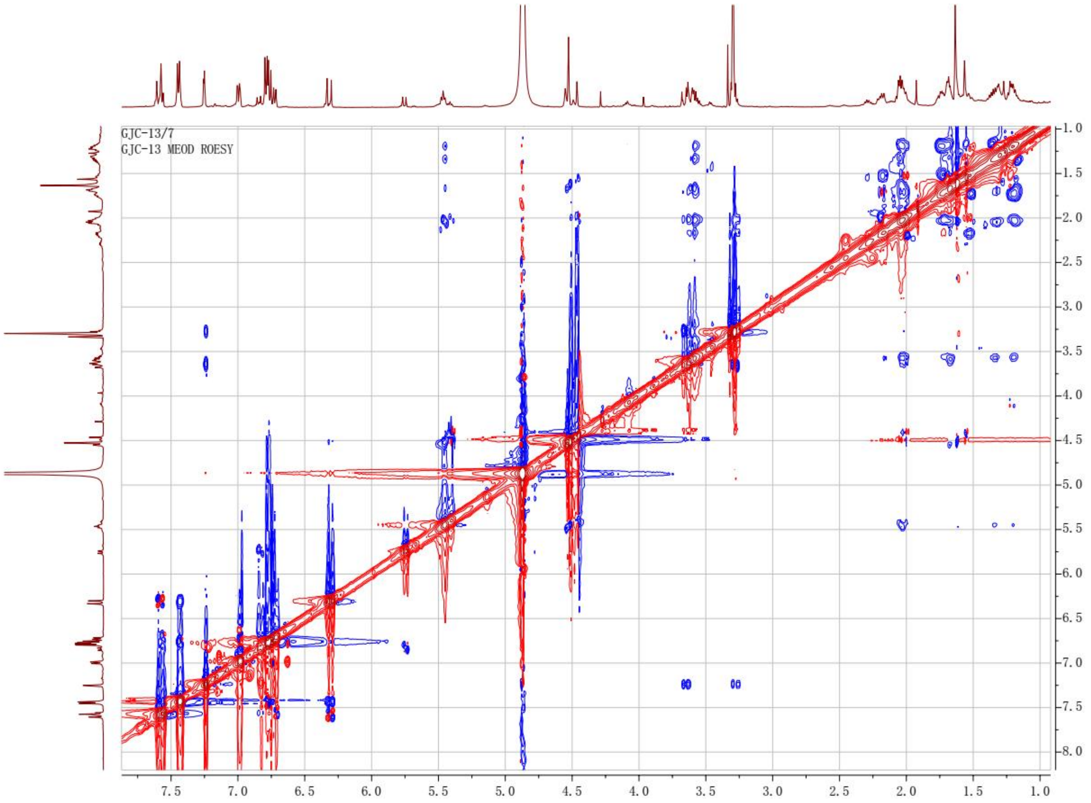

Figure S44. The HRESIMS Spectroscopic Data of Compound **5**

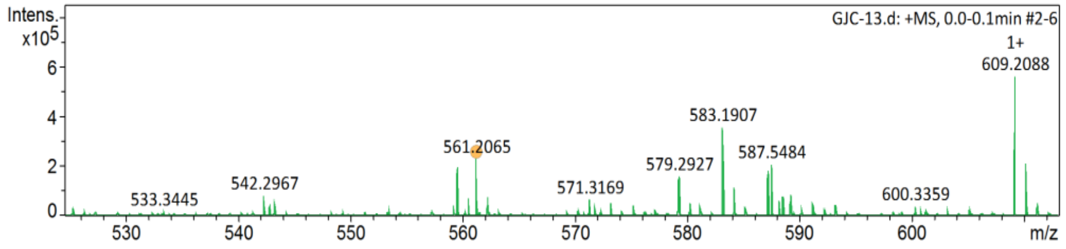

Meas. m/z	#	Ion Formula	m/z	err [ppm]	Mean err [ppm]	rdb	N-Rule	e⁻ Conf	mSigma	Adduct
561.206453	1	C30H34NaO9	561.209503	5.4	6.4	14.0	ok	even	14.6	M+Na

Figure S45. The IR Spectrum of Compound **5**

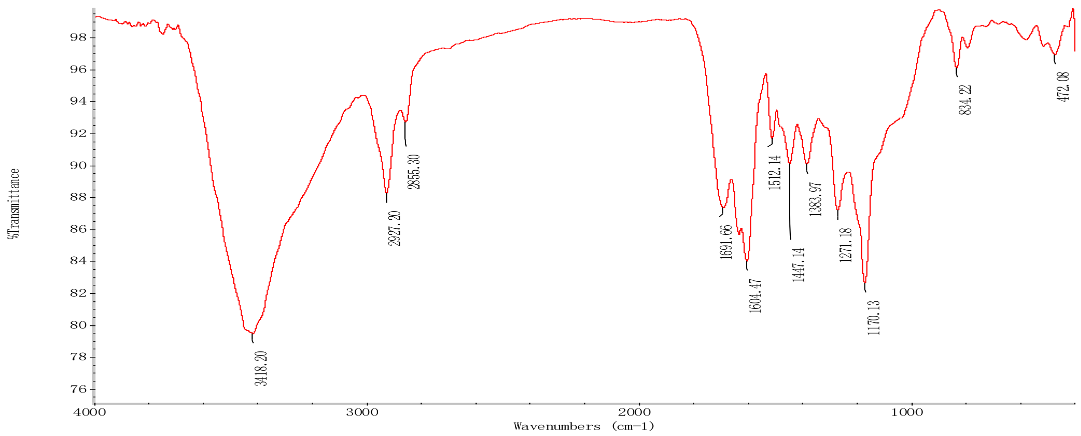

Figure S46. The ^{1}H NMR Spectrum of Compound **6** in CD_3OD

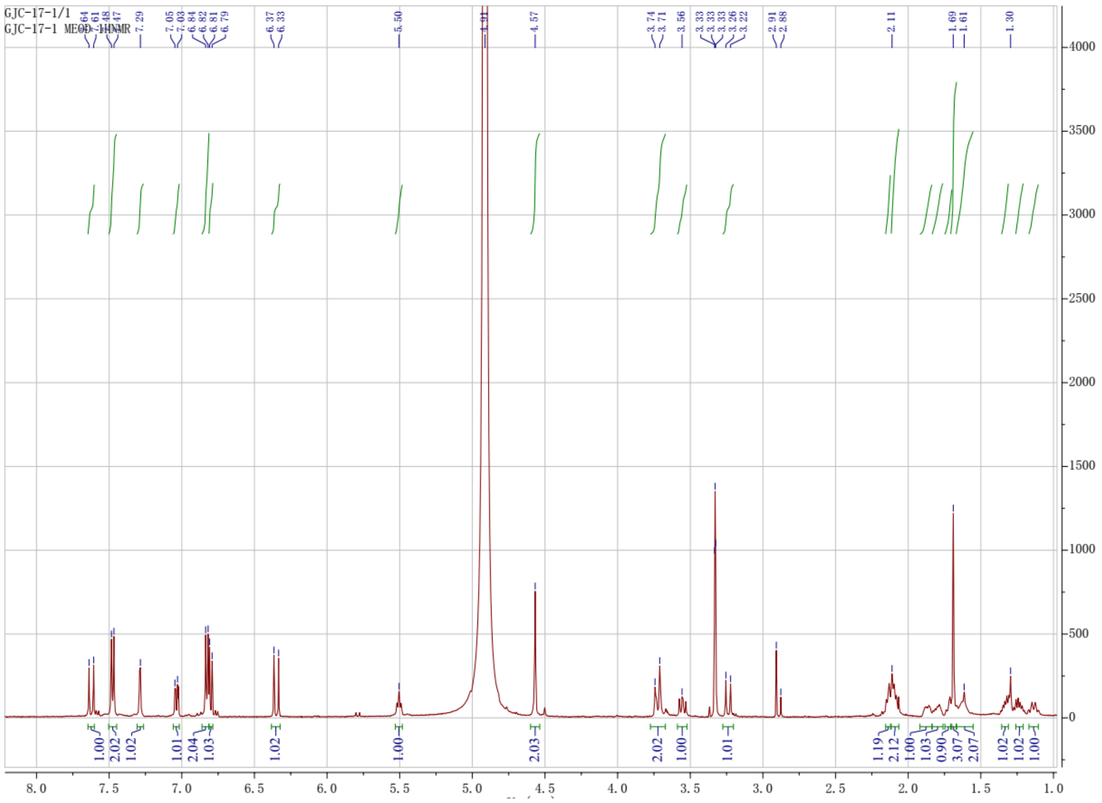

Figure S47. The ^{13}C NMR Spectrum of Compound **6** in CD_3OD

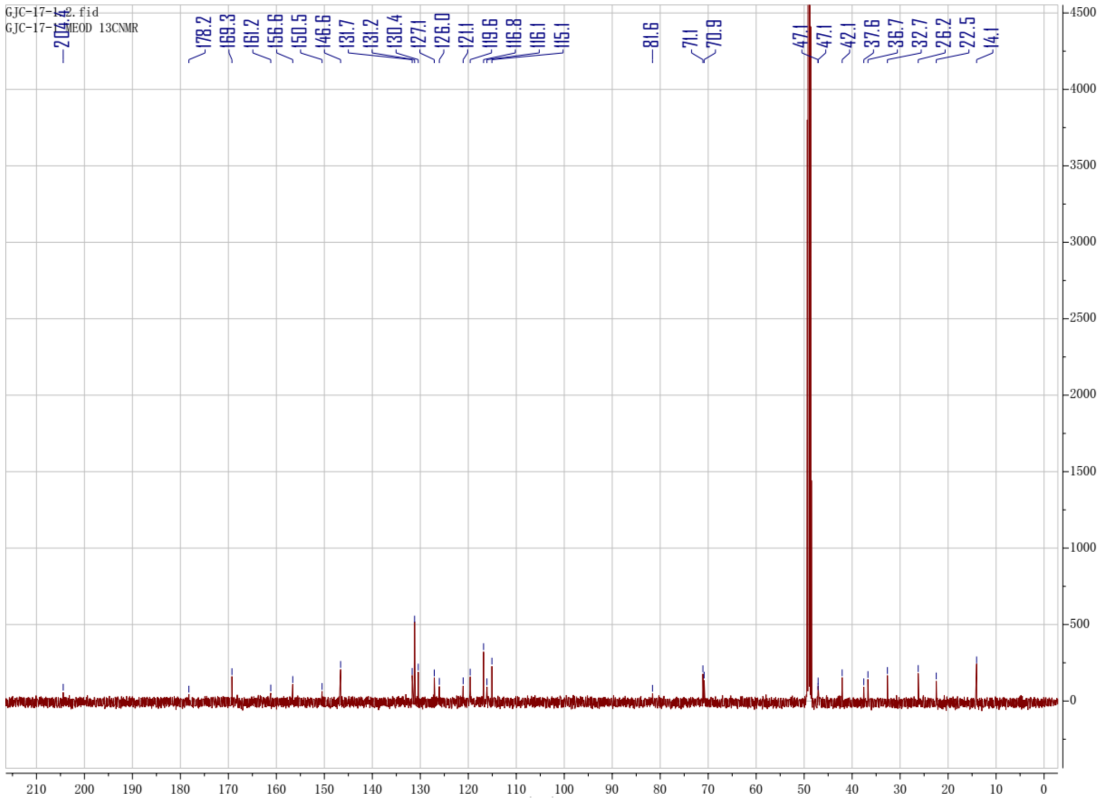

Figure S48. The DEPT Spectrum of Compound **6** in CD_3OD

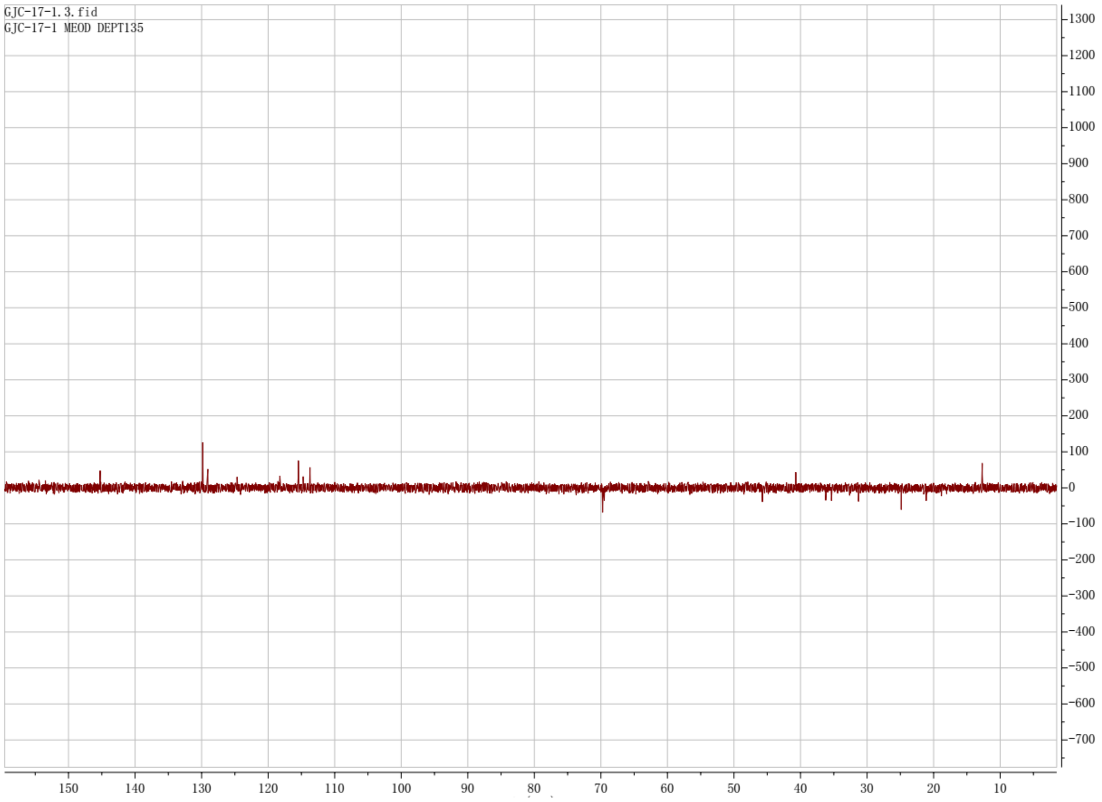

Figure S49. The HMQC Spectrum of Compound **6** in CD_3OD

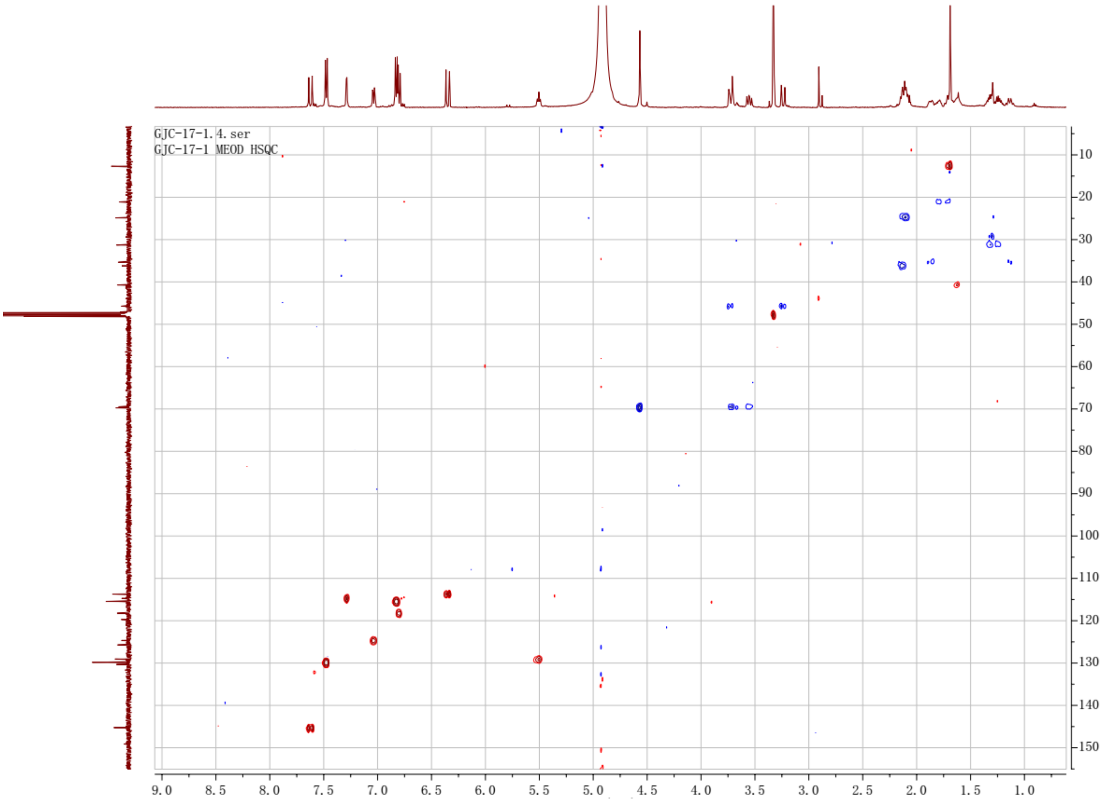

Figure S50. The HMBC Spectrum of Compound **6** in CD_3OD

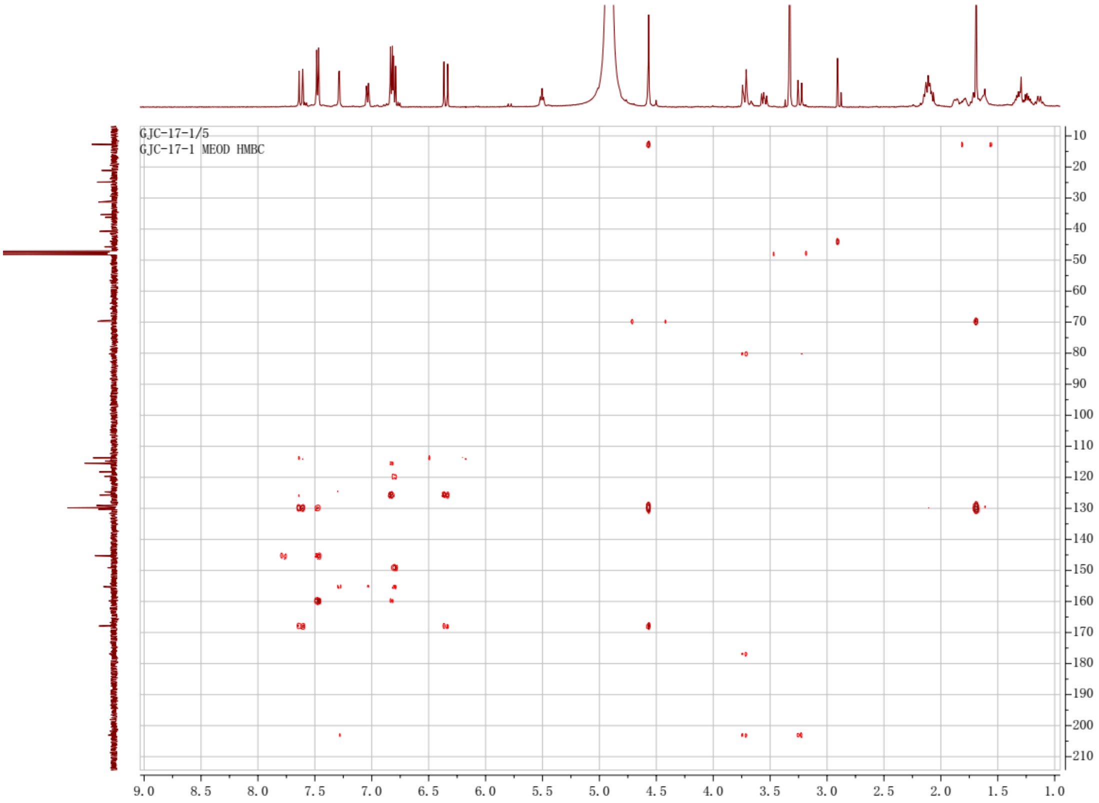

Figure S51. The COSY Spectrum of Compound **6** in CD_3OD

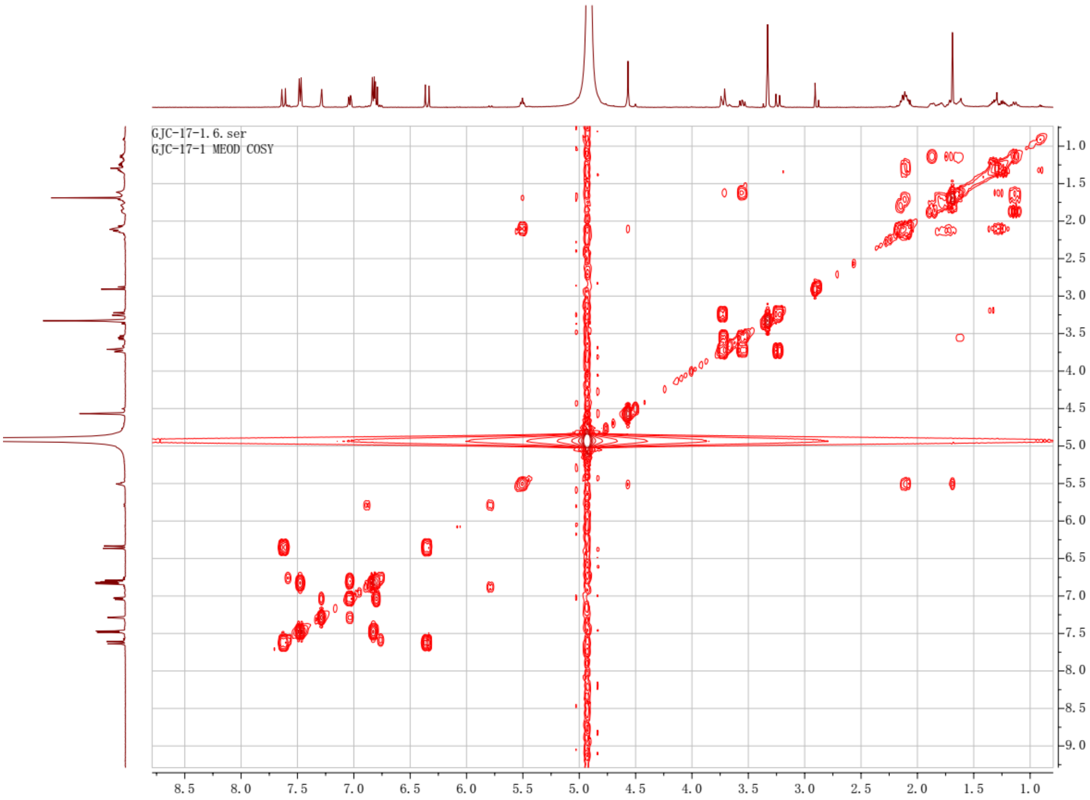

Figure S52. The ROESY Spectrum of Compound **6** in CD_3OD

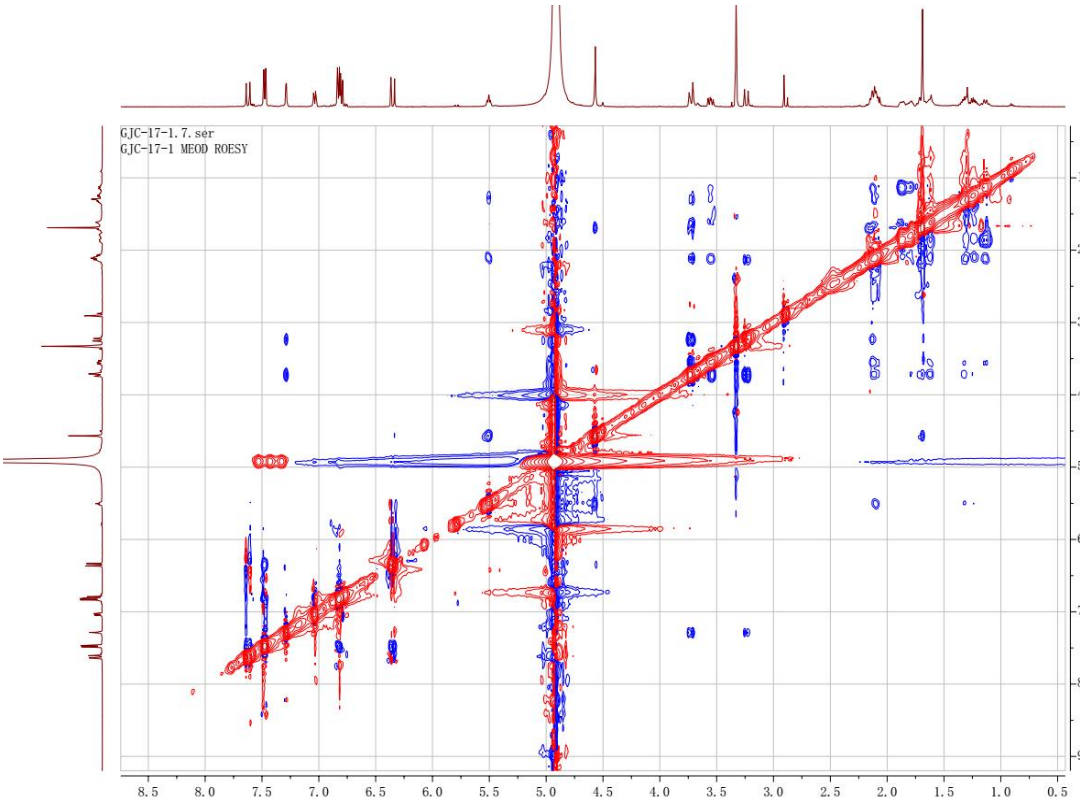

Figure S53. The HRESIMS Spectroscopic Data of Compound **6**

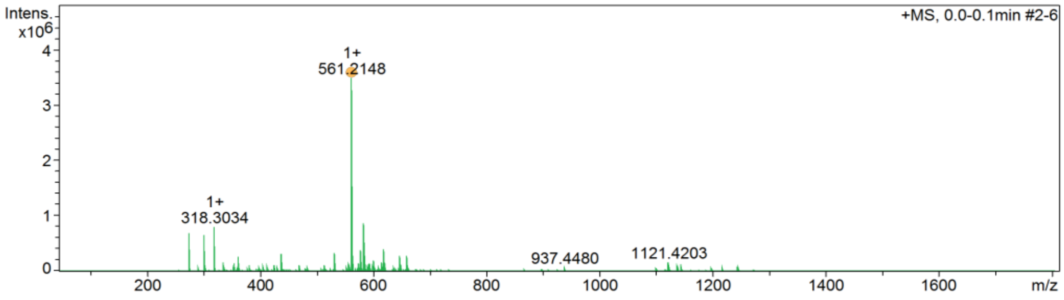

Meas. m/z	#	Ion Formula	m/z	err [ppm]	Mean err [ppm]	rdb	N-Rule	e¯ Conf	mSigma	Std I	Std Mean m/z	Std I VarNorm	Std m/z Diff	Std Comb Dev	Adduct
561.214794	1	C30H34NaO9	561.209503	-9.4	-9.1	14.0	ok	even	12.9	21.5	n.a.	n.a.	n.a.	n.a.	M+Na

Figure S54. The IR Spectrum of Compound **6**

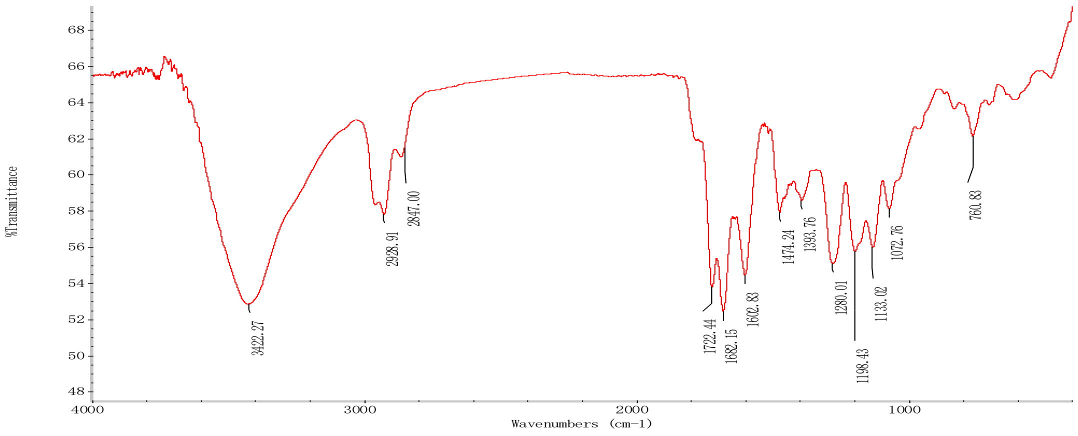

Figure S55. The ^{1}H NMR Spectrum of Compound **7** in CD_3OD

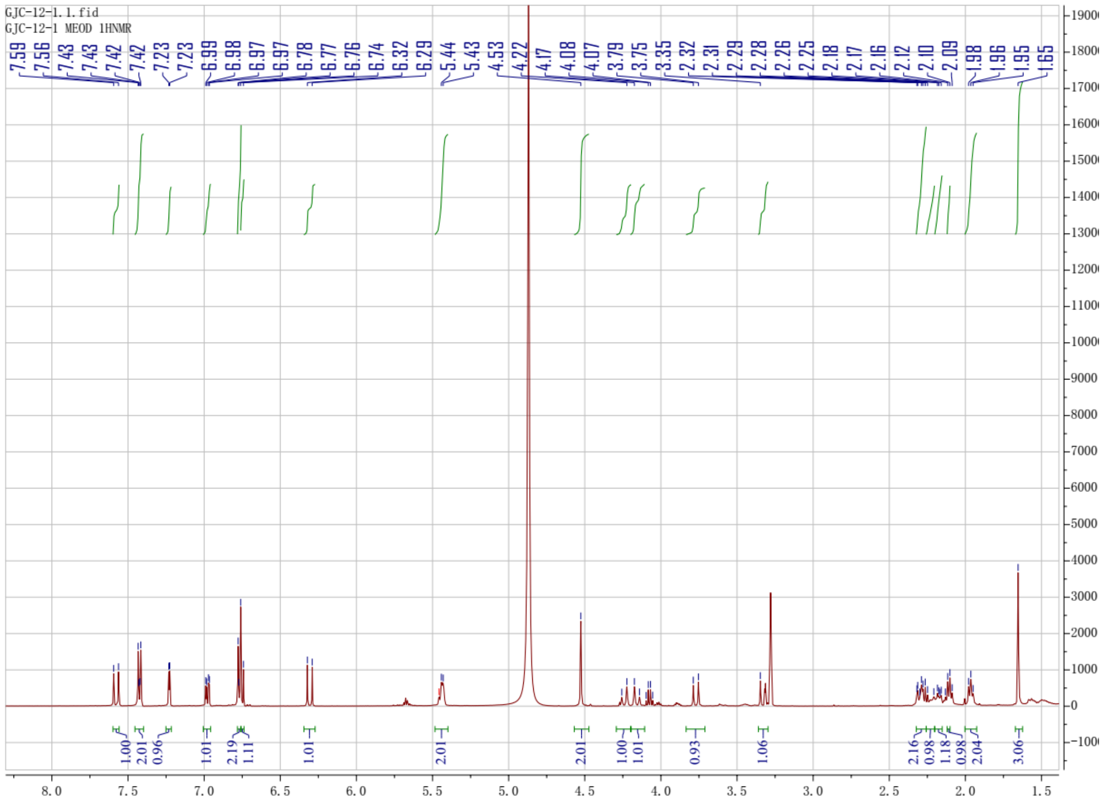

Figure S56. The ^{13}C NMR Spectrum of Compound **7** in CD_3OD

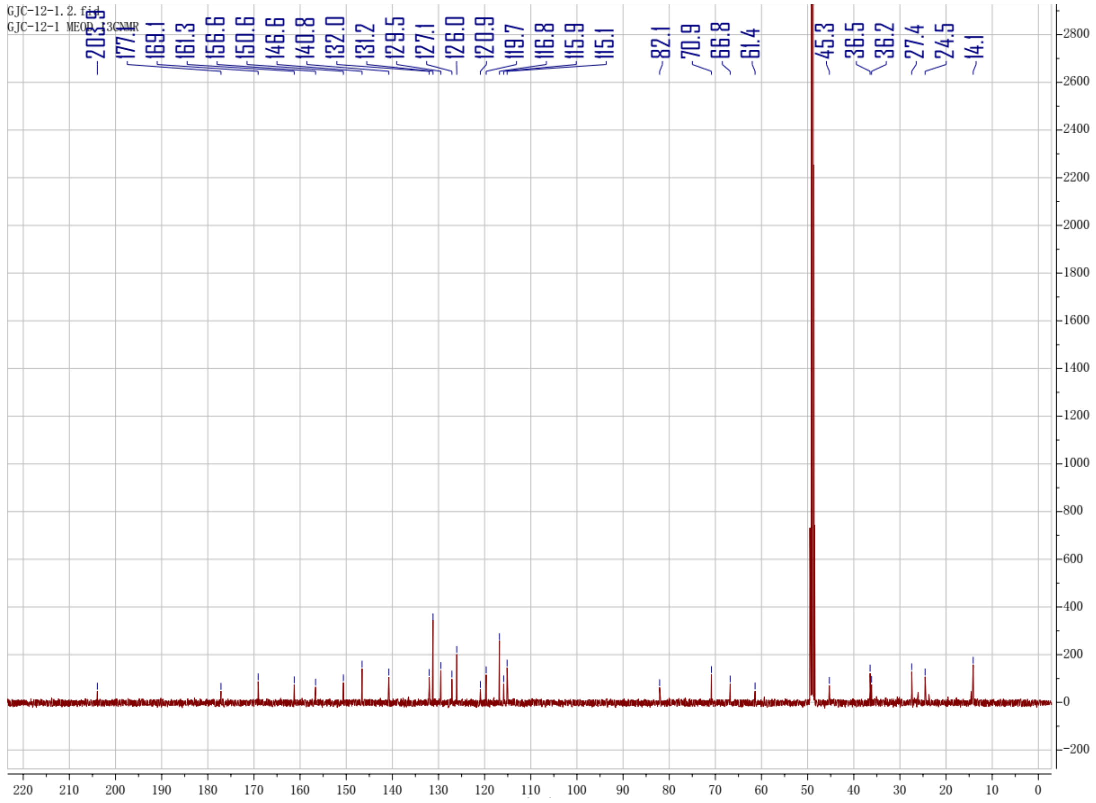

Figure S57. The DEPT Spectrum of Compound **7** in CD_3OD

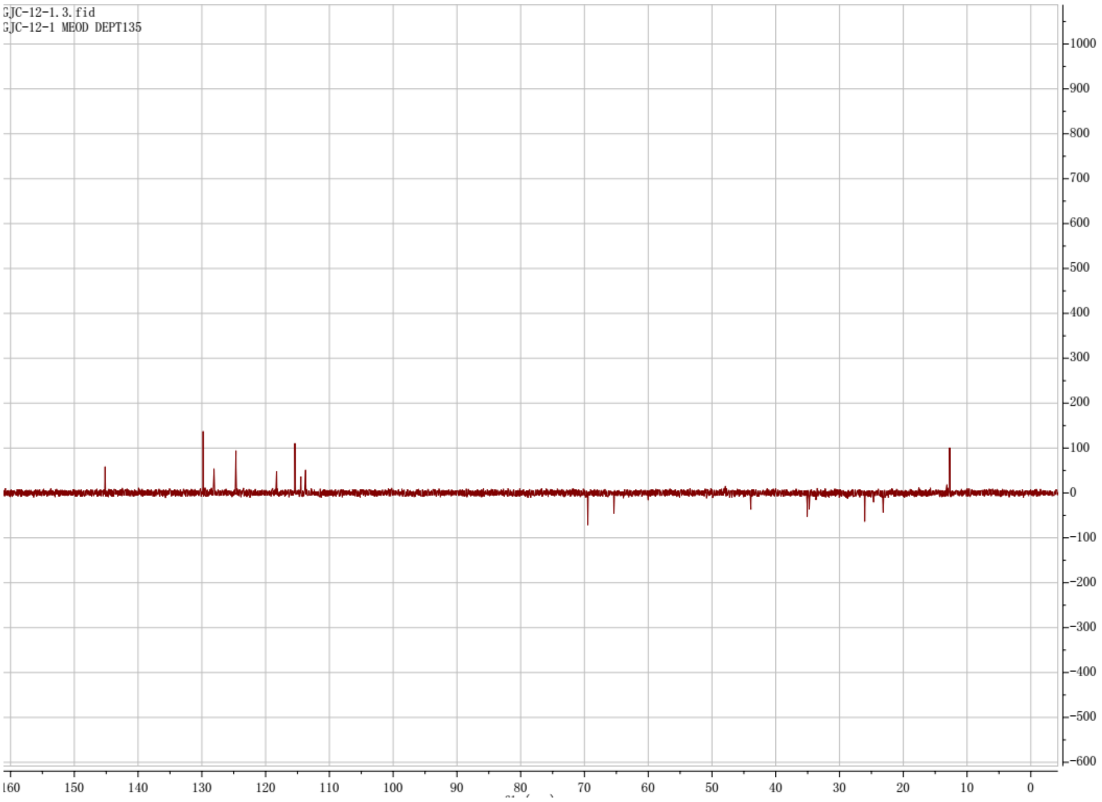

Figure S58. The HMQC Spectrum of Compound **7** in CD_3OD

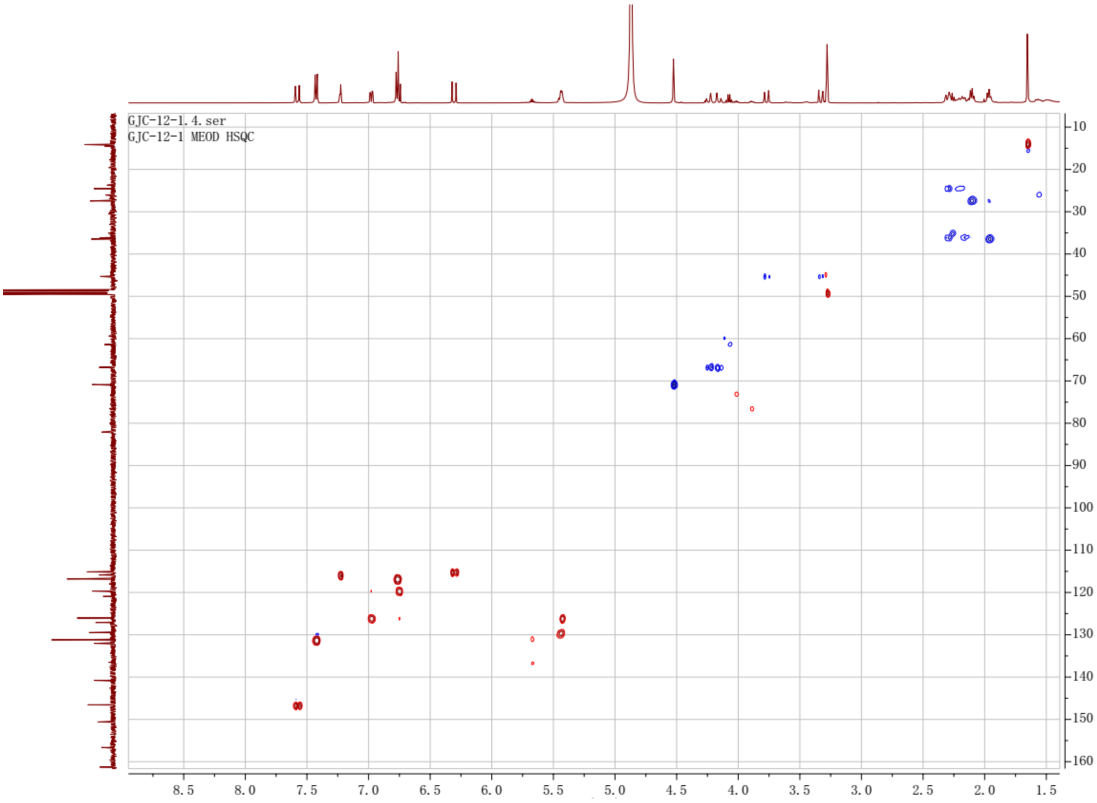

Figure S59. The HMBC Spectrum of Compound **7** in CD_3OD

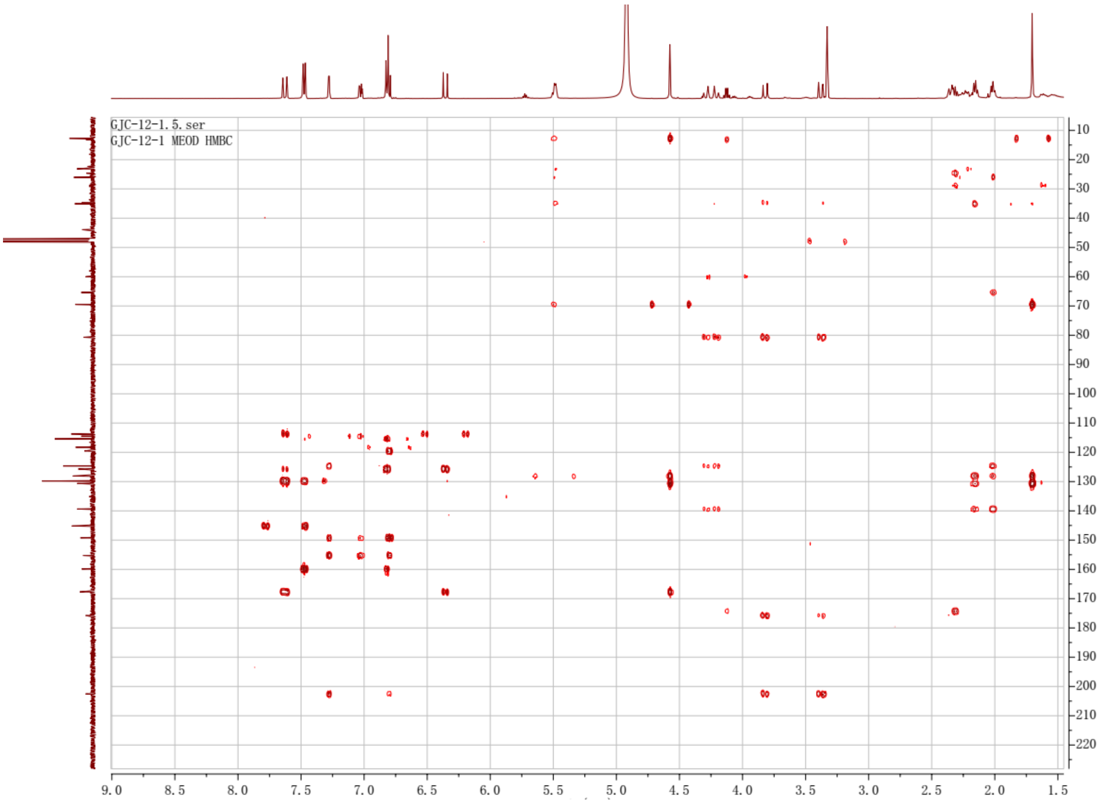

Figure S60. The COSY Spectrum of Compound **7** in CD_3OD

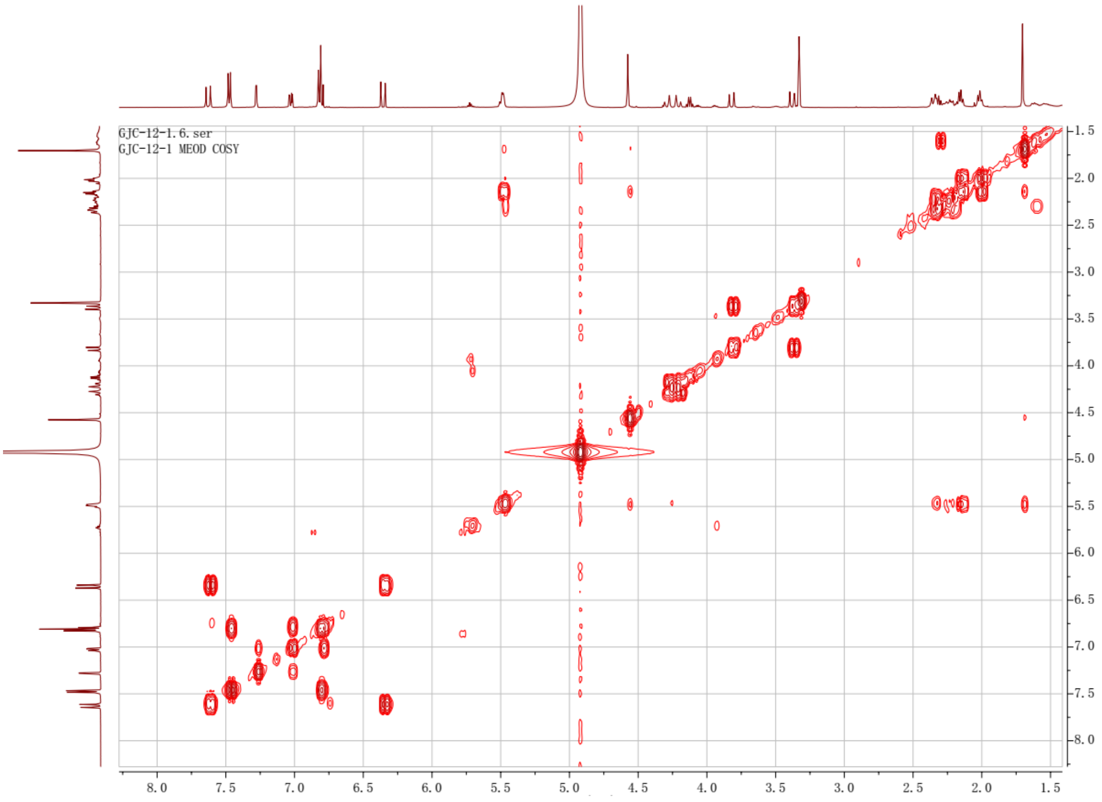

Figure S61. The ROESY Spectrum of Compound **7** in CD_3OD

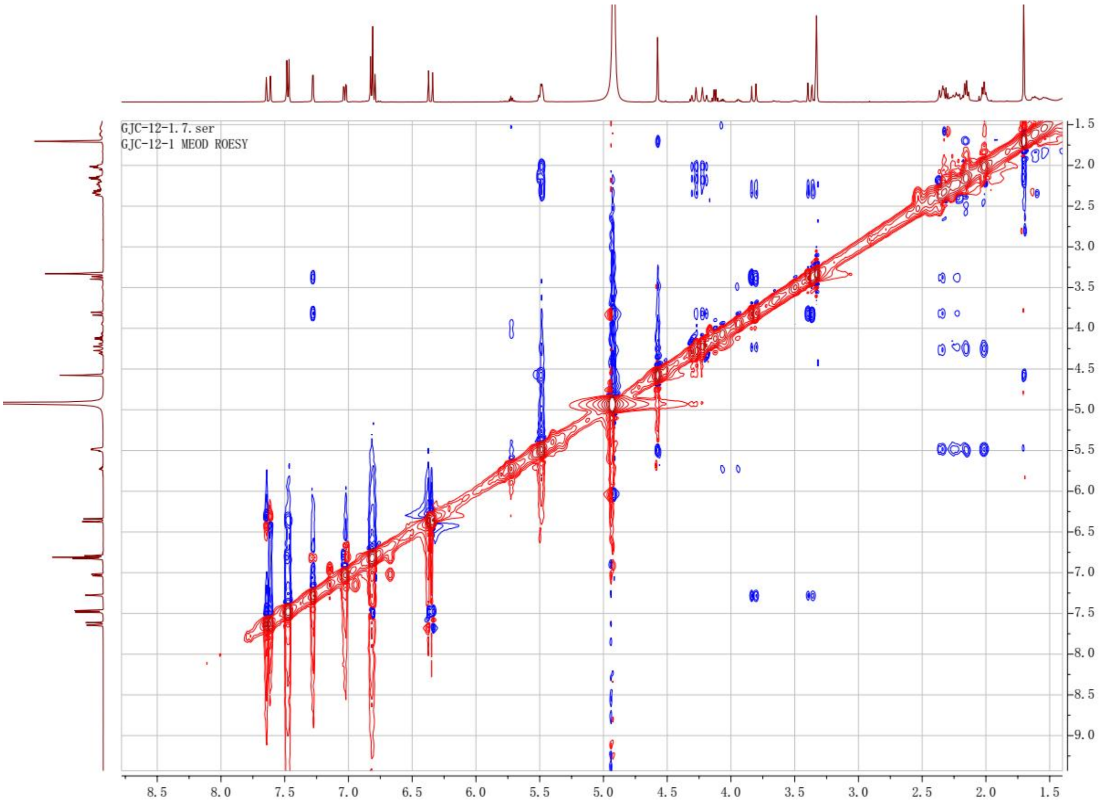

Figure S62. The HRESIMS Spectroscopic Data of Compound **7**

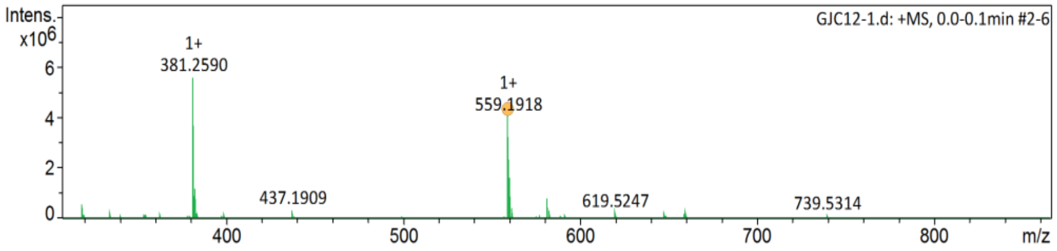

Meas. m/z	#	Ion Formula	m/z	err [ppm]	Mean err [ppm]	rdb	N-Rule	e⁻ Conf	mSigma	Adduct
559.191818	1	C30H32NaO9	559.193853	3.6	3.2	15.0	ok	even	33.8	M+Na

Figure S63. The IR Spectrum of Compound **7**

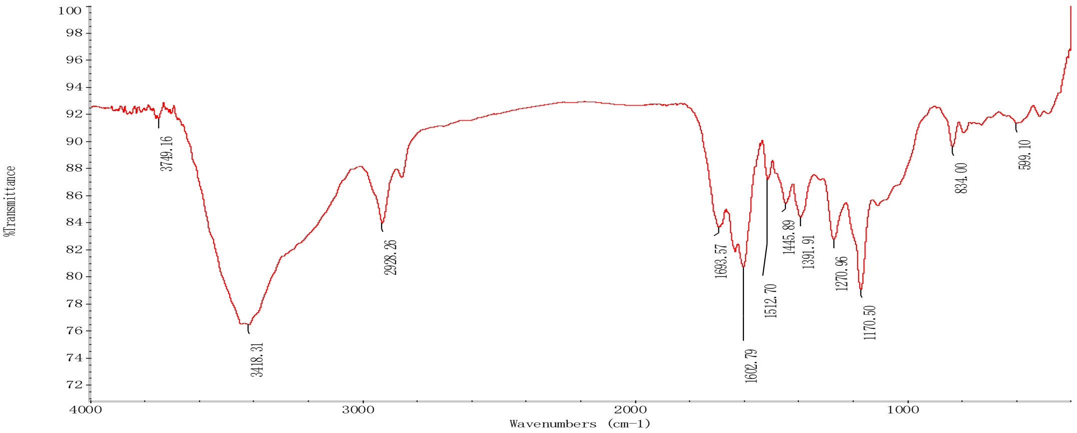

Figure S64. Inhibition rates of compounds **1-4** against PTP1B

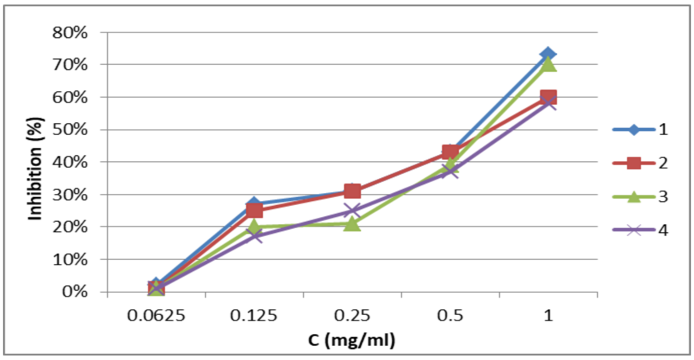

Supplement: Supplementary file 1 [file Data_Sheet_1.PDF]
